# Experimental evidence for electroacupuncture in managing pain from chemotherapy-induced peripheral neuropathy: a systematic review and meta-analysis

**DOI:** 10.1590/1414-431X2026e15562

**Published:** 2026-10-30

**Authors:** Ying Kong, Lei You, Mengwan Hu, Qiyuan Tian, Hong Sun, Kai Liu, Pengfei Li, Ping Song, Jingang Li

**Affiliations:** 1 The Second Affiliated Hospital of Heilongjiang University of Chinese Medicine, Harbin, China; 2 Heilongjiang Academy of Chinese Medicine Sciences, Harbin, China; 3 Graduate School, Heilongjiang University of Chinese Medicine, Harbin, China; 4 The First Affiliated Hospital of Heilongjiang University of Chinese Medicine, Harbin, China

**Keywords:** Electroacupuncture, CIPN, Pain, Meta-analysis, Mechanism

## Abstract

This study systematically evaluated the evidence from animal experiments on electroacupuncture (EA) for chemotherapy-induced peripheral neuropathy (CIPN) pain models. A systematic review and meta-analysis was conducted using the PICOS framework to assess the behavioral and molecular effects of EA and clarify its underlying mechanisms and translational potential. Thirteen eligible studies involving 235 animals were included. Methodological quality scores ranged from 5 to 8, with a mean of 6.5. Meta-analysis showed that EA significantly increased the paw withdrawal threshold (PWT), thermal pain withdrawal latency (PWL), and nerve conduction velocity (NCV), while reducing cold allodynia. At the molecular level, EA significantly decreased the expression of pro-inflammatory and sensitization markers, including TNF-α, IL-1β, GFAP, TLR4, TRPA1, and TRPV1, and increased the oxidative stress regulator Nrf2. Sensitivity analysis, publication bias assessment, subgroup analysis, and meta-regression were performed for the highly heterogeneous PWT outcome. Significant publication bias was identified and may account for much of the observed heterogeneity. Sensitivity analysis for TRPV1 suggested that this result may be disproportionately influenced by individual studies and should therefore be interpreted cautiously. Heterogeneity in the remaining outcomes likely reflects differences in animal models, acupoint selection, treatment duration, and intervention protocols. Overall, EA demonstrated analgesic and neuroprotective effects in CIPN models, primarily through modulation of neuroinflammation, attenuation of oxidative stress, and reduction of neuronal sensitization. These findings provide systematic preclinical evidence supporting EA as a promising adjuvant therapy for CIPN pain and highlight the need for high-quality clinical trials to optimize treatment protocols and facilitate clinical translation.

## Introduction

Pain associated with chemotherapy-induced peripheral neuropathy (CIPN) is a common and clinically challenging dose-limiting toxicity of cancer treatment. The incidence of CIPN can reach up to 68.1% within the first month following chemotherapy (1). Numerous widely used antineoplastic agents, including platinum-based compounds, taxanes, vinca alkaloids, and proteasome inhibitors, are known to induce CIPN to varying degrees (2). Painful sensory abnormalities, notably characterized by spontaneous tingling, burning pain, mechanical allodynia, or cold hyperalgesia, constitute a primary symptom. These painful sensations often coexist with sensory loss and numbness, significantly compromising patient adherence to treatment and overall quality of life (3,4).

Currently, clinical management options for CIPN-related pain remain severely limited. Mild pain is often managed with symptomatic medications, while moderate to severe pain frequently necessitates dose reduction or discontinuation of chemotherapy, potentially compromising cancer control outcomes. Existing therapeutic approaches are not only insufficient in efficacy but also often provide delayed and incomplete symptom relief, leading to persistent pain and the development of chronic neuropathic pain conditions. Although the American Society of Clinical Oncology (ASCO) guidelines recommend duloxetine for the treatment of painful CIPN (5), its effectiveness varies significantly among individuals, and side effects such as nausea and headache limit its long-term use. Therefore, focusing on the pathological mechanisms underlying CIPN-associated pain and exploring safe and effective intervention strategies hold significant scientific value and clinical urgency.

Electroacupuncture (EA) is a modern acupuncture technique that combines traditional acupuncture with electrical stimulation. Compared with manual needling, EA offers the advantage of standardized and consistently maintainable stimulation parameters, enabling quantifiable and controllable intervention (6). This technical feature allows EA to produce more defined and reproducible physiological effects, demonstrating unique potential in the management of neuropathic pain (7).

Previous studies have shown that EA exerts clear analgesic effects in various animal models of neuropathic pain - such as spinal nerve ligation (L5/L6), chronic constriction injury (CCI), and spared nerve injury (SNI), manifested as increased mechanical threshold and reduced thermal hyperalgesia (8-10). These findings provide preliminary mechanistic insights into EA's role in neuropathic pain treatment. However, regarding CIPN as a specific subtype of neuropathic pain, comprehensive evidence for the overall efficacy of EA and its systematic mechanisms of action has not yet been thoroughly evaluated. Therefore, this study aimed to systematically review the interventional effects of EA in animal models of CIPN and synthesize related mechanistic evidence. This comprehensive assessment will help clarify the potential role and biological basis of EA in CIPN pain management, thereby providing a valuable reference for future translational research and clinical application.

## Methods

### Research and registration

This study was conducted and reported in strict accordance with the Cochrane and PRISMA (Preferred Reporting Items for Systematic Reviews and Meta-Analyses) guidelines (11). The study protocol was prospectively registered on PROSPERO (Registration No. CRD420251272055).

### Inclusion and exclusion criteria

#### Inclusion criteria based on PICOS

P: Animal studies utilizing rat or mouse models (all sexes). I: Electroacupuncture (EA) as the sole intervention. C: Models of CIPN, including paclitaxel-induced (PIPN), oxaliplatin-induced (OIPN), and bortezomib-induced peripheral neuropathy (BIPN). O: Outcomes: The primary outcome measure was paw withdrawal threshold (PWT). Secondary outcomes included behavioral assessments (thermal pain withdrawal latency (PWL), cold allodynia), physiological indicators (nerve conduction velocity (NCV), body weight gain rate), neuroinflammatory markers (tumor necrosis factor (TNF)-α, interleukin (IL)-1β, GFAP, TLR4), neuronal sensitization markers (TRPA1, TRPV1), and oxidative stress markers (Nrf2). S: All randomized controlled trials investigating the effects of EA on animal models of chemotherapy-induced peripheral neuropathy.

#### Exclusion criteria

Studies were excluded based on the following criteria: 1) non-exclusive EA intervention, such as EA combined with other acupuncture modalities or pharmacological agents; 2) case reports, clinical trials, crossover studies, reviews, meta-analyses, dissertations, theses, or conference abstracts; 3) studies not focused on CIPN or non-animal experimental research; 4) insufficient or incomplete data for definitive outcome assessment; 5) Duplicate publications or studies deemed methodologically unsound; 6) None of the pain-related outcome measures.

### Data extraction

Two investigators independently performed literature screening and data extraction. Following this process, cross-verification was conducted. Any discrepancies were resolved by consulting a third researcher, and final inclusion was determined upon achieving consensus among all three. Literature screening was performed using EndNote (version X9). Data extraction was aided by GetData Graph Digitizer (version 2.26) for digitizing graphical data. When significant heterogeneity was observed for a specific outcome, data transformation was attempted; studies were excluded if data could not be transformed and pooled. Prior to extraction, a preliminary data extraction template was developed based on the study objectives and inclusion criteria. Extracted parameters included: study characteristics (author, publication year, country, model type, animal species); EA protocol (acupoint selection, stimulation frequency, session duration, treatment period); and outcome measures (behavioral, physiological status, neuroinflammation, neuronal sensitization, oxidative stress). To validate the template, a pilot test was conducted on a random subset comprising 20% of the included studies.

### Quality assessment

The risk of bias was assessed using the SYRCLE's risk of bias (ROB) tool (12), with each of its ten domains judged as having a low, unclear, or high risk of bias. Methodological quality was evaluated using the CAMARADES checklist (13), which scores studies against ten predefined criteria. An overall mean score was calculated to quantify study quality. Any discrepancies in assessments were resolved through consensus discussion with a third investigator. Detailed assessment criteria are provided in the Supplementary Materials (A-G).

### Data analysis

Statistical analyses were performed using Stata software (version 15.0), including forest plot generation, sensitivity analysis, assessment of publication bias, funnel plot analysis, subgroup analysis, and meta-regression. Continuous outcome variables were analyzed using the mean difference (MD) or standardized mean difference (SMD) as effect measures. Heterogeneity was quantified with the I^2^ statistic. If the included studies were homogeneous in terms of animal model, age, sex, and housing conditions, or if *I* ^2^≤50%, a fixed-effects model was applied; otherwise, a random-effects model (inverse variance method) was used. High heterogeneity was addressed through sensitivity analysis, in which each study was excluded sequentially to identify sources of variation, supplemented by stratified subgroup analysis and meta-regression to explore residual heterogeneity. For primary outcomes with 10 or more included studies, publication bias was evaluated using Egger's test, Begg's test, funnel plot asymmetry, and the trim-and-fill method.

## Results

### Literature search and screening process

Based on the pre-defined MeSH-based search strategy, a total of 408 relevant records were identified from database inception until November 23, 2025. After removing 60 duplicate records, 348 studies underwent title and abstract screening, resulting in the exclusion of 270 irrelevant records. One full-text article was unavailable, and one was retracted on June 27, 2026. Following full-text assessment of the remaining 76 articles, 63 were excluded for not meeting the inclusion/exclusion criteria. Consequently, 13 studies were included in the final meta-analysis. The literature searches and screening process are detailed in Figure 1. The basic characteristics of the 13 included studies are summarized in Table 1 (14-26).

**Figure 1:**
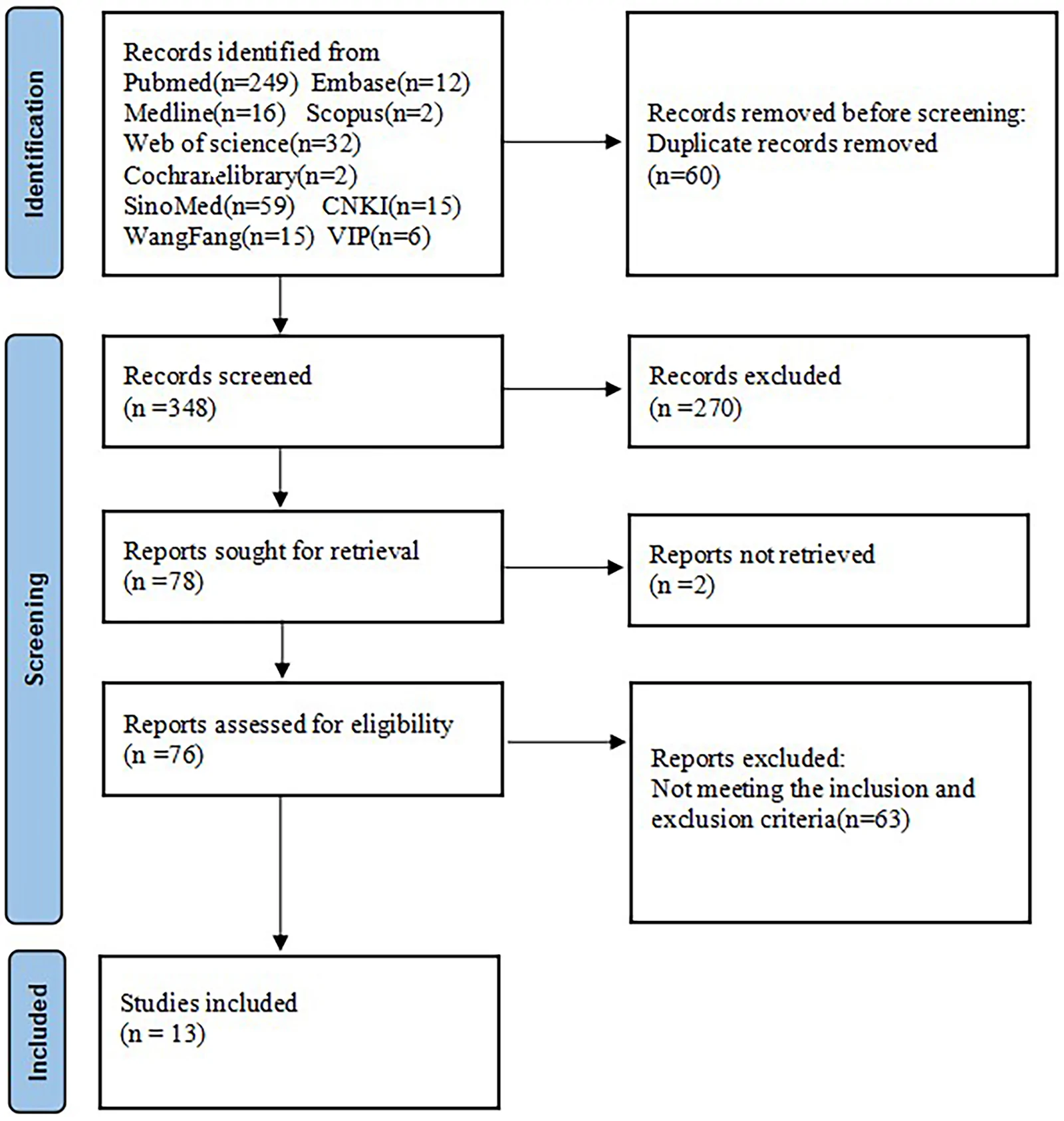
Literature search and screening process.

**Table 1:** List of research information.

Author	Country	Animal models	N	Acupuncture measures				Current	Outcome indicators	Scores
				Acupuncture location	Frequency	Time	Cot			
Zhang et al. 14	China	PIPN/Mouse	16	GB30	QD	30 min	7 d	1 mA 2 Hz	① ⑦	7
Ye et al. 15	China	OPIN/Rat	10	ST36	QD	10 min	14 d	1 mA 5 Hz	① ④ ⑤ ⑧	6
Shi et al. 16	China	OPIN/Rat	20	ST36	QOD	30 min	14 d	1 mA 2 Hz	① ③ ⑩	5
Pang 17	China	PIPN/Mouse	24	ST36	QOD	15 min	14 d	0.5 mA 10 Hz	① ② ⑥	7
Li et al. 18	China	PIPN/Rat	14	ST36 BL60	QD	30 min	9 d	0.5-1.5 mA 2 Hz	① ⑪	6
Cui et al. 19	China	OPIN/Rat	20	ST36	QD	30 min	5 d	2 mA 2 Hz	④	6
Zhao et al. 20	China	PIPN/Rat	15	ST36	QOD	30 min	14 d	1mA 10 Hz	① ⑤ ⑥ ⑦ ⑧ ⑨	6
Zhao et al. 21	China	PIPN/Rat	27	PC5 PC6	QD	20 min	5 d	2 mA 2 Hz	① ② ⑥ ⑦ ⑫	8
Wang et al. 22	China	PIPN /Rat	15	ST36	QOD	30 min	14 d	1 mA 2 Hz	① ③ ⑩ ⑪ ⑫	7
Li et al. 23	China	PIPN/Rat	10	ST36 BL60	QOD	30 min	7 d	0.5 mA 2 Hz	① ② ⑤ ⑨ ⑪	7
Li et al. 24	China	PIPN/Rat	10	ST36 BL60	QOD	30 min	7 d	0.5 mA 2 Hz	①	6
Choi et al. 25	Korea	PIPN/Mouse	22	ST36	QOD	30 min	14 d	2 mA 2 Hz	① ②	8
Chen et al. 26	China	PIPN/Mouse	32	ST36	QD	20 min	4d	1 mA 2 Hz	① ⑧	6

①PWT ②PWL ③Cold allodynia ④NCV ⑤Weight gain ⑥TNF-α ⑦IL-1β ⑧GFAP ⑨TLR4 ⑩TRPA1 ⑪TRPV1 ⑫Nrf2. PWT: paw withdrawal threshold; PWL: thermal pain withdrawal latency; NCV: nerve conduction velocity; TNF-α: tumor necrosis factor-α; IL-1β: interleukin-1β; GFAP: glial fibrillary acidic protein; TLR4: toll-like receptor 4; TRAP1: tumor necrosis factor receptor-associated protein 1; TRPV1: transitional receptor potential vanilloid 1; Nrf2: nuclear factor-erythroid-2-related factor 2; N: sample size; QD: once daily; QOD: once every other day; d: Day; Cot: course of treatment; Scores: Quality scores of included studies (5-8, mean 6.5).

### Assessment of risk of bias and methodological quality

The risk of bias for the 13 included studies was evaluated using the SYRCLE's ROB tool. Individual study scores ranged from 6 to 9, with a mean score of 6.5. Detailed domain-specific assessments are presented in Table 2 (14-26). Methodological quality was assessed using the CAMARADES checklist. Scores for individual studies ranged from 5 to 8, yielding a mean score of 6.5. The complete quality assessment details are provided in Table 3 (14-26).

**Table 2:** Assessment of risk of bias.

Study	1	2	3	4	5	6	7	8	9	10	Scores
Zhang et al. 14	L	L	?	L	NA	L	L	L	L	L	8
Ye et al. 15	L	L	?	L	NA	?	?	L	L	L	6
Shi et al. 16	L	L	L	L	NA	?	?	L	L	L	7
Pang 17	L	L	L	L	NA	L	L	L	L	L	9
Li et al. 18	L	L	?	L	NA	?	?	L	L	L	6
Cui et al. 19	L	L	?	L	NA	?	?	L	L	L	6
Zhao et al. 20	L	L	?	L	NA	?	?	L	L	L	6
Zhao et al. 21	L	L	L	L	NA	?	L	L	L	L	8
Wang et al. 22	L	L	?	L	NA	?	?	L	L	L	6
Li et al. 23	L	L	?	L	NA	?	?	L	L	L	6
Li et al. 24	L	L	?	L	NA	?	?	L	L	L	6
Choi et al. 25	L	L	?	L	NA	?	L	L	L	L	7
Chen et al. 26	L	L	?	L	NA	?	?	L	L	L	6

L: low risk of bias; H: high risk of bias; ?: unclear risk of bias; NA: not applicable.

**Table 3:** Quality assessment of included studies.

Author	1	2	3	4	5	6	7	8	9	10	Scores
Zhang et al. 14	Y	Y	Y	?	Y	Y	Y	?	Y	?	7
Ye et al. 15	Y	Y	Y	?	?	Y	Y	?	Y	?	6
Shi et al. 16	Y	?	Y	?	?	Y	Y	?	Y	?	5
Pang 17	Y	Y	Y	?	Y	Y	Y	?	Y	?	7
Li et al. 18	Y	Y	Y	?	?	Y	Y	?	Y	?	6
Cui et al. 19	Y	Y	Y	?	?	Y	Y	?	Y	?	6
Zhao et al. 20	Y	Y	Y	?	?	Y	Y	?	Y	Y	6
Zhao et al. 21	Y	Y	Y	?	Y	Y	Y	?	Y	Y	8
Wang et al. 22	Y	Y	Y	?	?	Y	Y	?	Y	Y	7
Li et al. 23	Y	Y	Y	?	?	Y	Y	?	Y	Y	7
Li et al. 24	Y	Y	Y	?	?	Y	Y	?	Y	?	6
Choi et al. 25	Y	Y	Y	?	Y	Y	Y	?	Y	Y	8
Chen et al. 26	Y	Y	Y	?	?	?	Y	?	Y	Y	6

Y: yes; N: no; ?: unclear.

### Meta-analysis results

The following results were derived from the meta-analysis, with detailed forest plots presented in Figure 2. More comprehensive forest plots have also been included in the Supplementary Materials for further reference.

**Figure 2:**
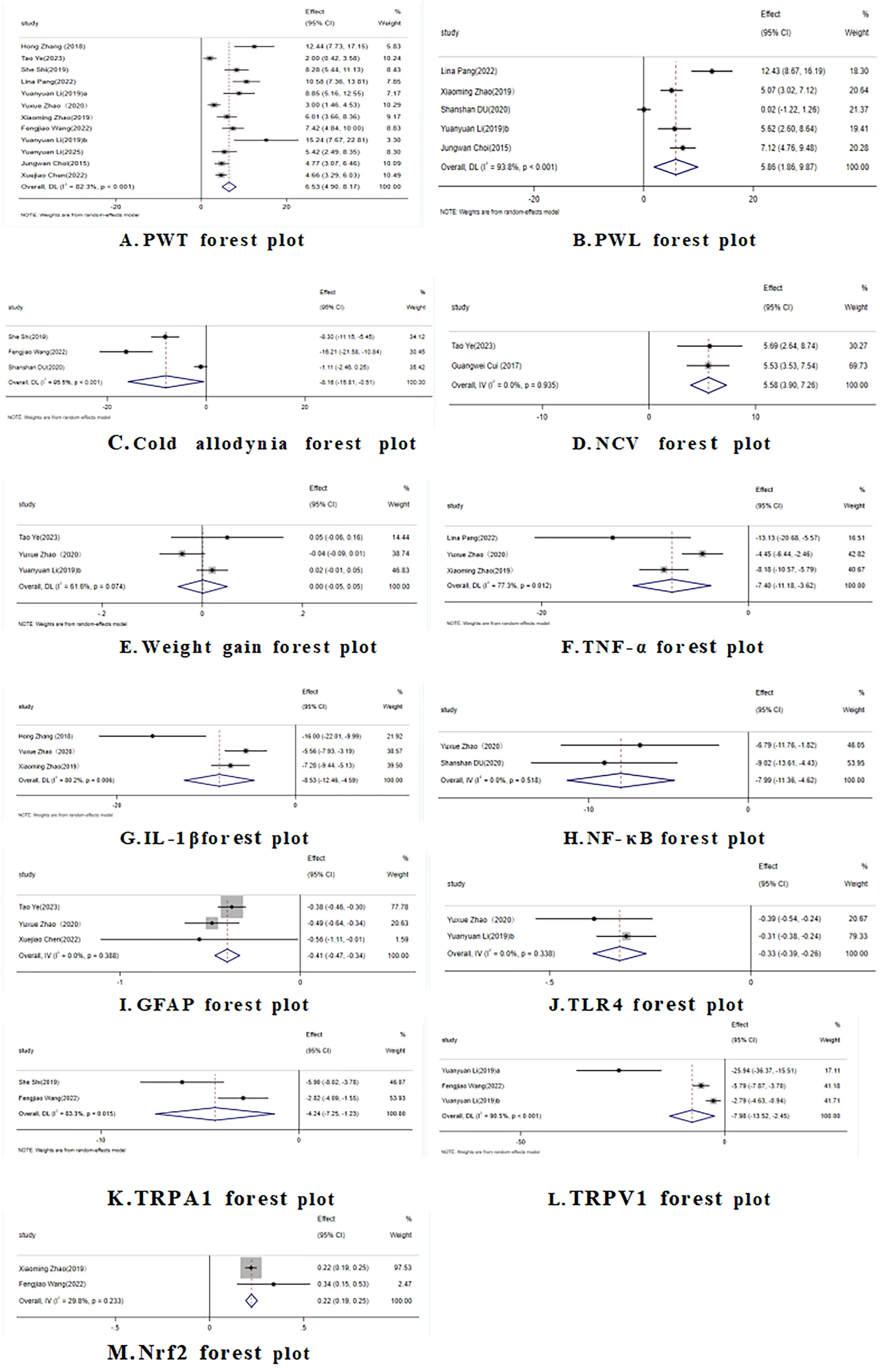
Forest plots of **A**, paw withdrawal threshold (PWT); **B**, thermal pain latency (PWL); **C**, cold allodynia; **D**, nerve conduction velocity; **E**, weight gain; **F**, tumor necrosis factor (TNF)-α; **G**, interleukin (IL)1β; **H**, glial fibrillary acidic protein (GFAP); **I**, toll-like receptor 4 (TLR4); **J**, tumor necrosis factor receptor-associated protein 1 (TRPA1); **K**, transitional receptor potential vanilloid 1 (TRPV1); and **L**, nuclear factor-erythroid-2-related factor 2 (Nrf2).

#### Behavioral outcomes

Twelve studies reported on the PWT, encompassing a total of 210 animal subjects. Heterogeneity testing indicated substantial heterogeneity (I^2^=82.3%, P<0.001). Consequently, a random-effects model was applied for the meta-analysis. The pooled effect was significant [SMD=6.553, 95%CI (4.899, 8.167), Z=7.836, P=0.000] (Figure 2A).

Four studies reported the PWL, involving data from 73 animals. The heterogeneity test revealed high heterogeneity (I^2^=97.4%, P<0.001), leading to the use of a random-effects model for the meta-analysis. The pooled effect was significant [SMD=3.958, 95%CI (2.801, 5.115), Z=6.705, P<0.001] (Figure 2B).

Two studies reported on cold allodynia, with data from 40 animals. The heterogeneity test indicated substantial heterogeneity (I^2^=84.6%, P=0.011). A random-effects model was therefore applied for the meta-analysis. The pooled effect was significant [SMD=-11.913, 95%CI (-19.638, -4.188), Z=-3.023, P=0.003] (Figure 2C).

#### Physiological indicators

Two studies reported NCV, involving data from 30 animals. The heterogeneity test indicated no heterogeneity (I^2^=0%, P=0.935). Consequently, a fixed-effects model was employed for the meta-analysis. The pooled effect was significant [MD=5.581, 95%CI (3.903, 7.259), Z=6.519, P<0.001] (Figure 2D).

Three studies reported weight gain, encompassing data from 35 animals. Heterogeneity testing indicated moderate heterogeneity (*I* ^2^ *=*61.6%, P=0.074). A random-effects model was therefore applied for the meta-analysis. The pooled effect size was not statistically significant [MD=0.001, 95%CI (-0.048, 0.050), *Z*=0.043, P=0.965] (Figure 2E).

#### Neuroinflammatory

Three studies reported data on TNF-α, comprising a total of 50 animals. Heterogeneity testing indicated substantial heterogeneity (I^2^=77.3%, P=0.012). Therefore, a random-effects model was applied. The pooled effect was significant [SMD=-7.400, 95%CI (-11.179, -3.621), Z=-3.838, P<0.001] (Figure 2F).

Three studies reported data on IL-1β, involving a total of 58 animals. The heterogeneity test indicated significant heterogeneity (I^2^=80.2%, P=0.006). Therefore, a random-effects model was employed for the meta-analysis. The pooled effect was significant [SMD=-8.530, 95%CI (-12.465, -4.595), Z=-4.249, P<0.001] (Figure 2G).

Three studies reported data on GFAP, involving a total of 22 animals. The heterogeneity test indicated no heterogeneity (I^2^=0%, P=0.388). A fixed-effects model was therefore employed for the meta-analysis. The pooled effect was significant [MD=-0.406, 95%CI (-0.475, -0.336), Z=-11.496, P<0.001] (Figure 2H).

Two studies reported data on TLR4, involving a total of 18 animals. The heterogeneity test indicated no heterogeneity (I^2^=0%, P=0.338). A fixed-effects model was therefore employed for the meta-analysis. The pooled effect was significant [MD=-0.327, 95%CI (-0.393, -0.260), Z=-9.662, P<0.001] (Figure 2I).

#### Neuronal sensitization

Two studies reported data on TRPA1, involving a total of 40 animals. The heterogeneity test indicated substantial heterogeneity (I^2^=83.3%, P=0.015). Consequently, a random-effects model was employed for the meta-analysis. The pooled effect was significant [SMD=-4.241, 95%CI (-7.250, -1.231), Z=-2.762, P=0.006] (Figure 2J).

Three studies reported data on TRPV1, involving a total of 44 animals. The heterogeneity test indicated high heterogeneity (I^2^=90.5%, P<0.001). A random-effects model was therefore applied for the meta-analysis. The pooled effect was significant [SMD=-7.985, 95%CI (-13.516, -2.454), Z=-2.830, P=0.005] (Figure 2K).

#### Oxidative stress

Two studies reported data on Nrf2, involving a total of 40 animals. The heterogeneity test indicated low heterogeneity (I^2^=29.8%, P=0.233). A fixed-effects model was therefore employed for the meta-analysis. The pooled effect was significant [MD=0.223, 95%CI (0.192, 0.254), Z=14.279, P<0.001] (Figure 2L).

### Sensitivity and subgroup analyses

Significant heterogeneity (*I* ^2^>50%) was observed across several outcomes: PWT, PWL, cold allodynia, weight gain, TNF-α, IL-1β, TRPA1, and TRPV1. A random-effects model was applied to account for this variability. To assess the robustness of our findings, sensitivity analyses were performed based on the risk of bias levels of the included studies. Sequential removal of individual studies revealed that the removal of certain studies on TRPV1 substantially influenced the overall heterogeneity estimates, while the results for all other outcomes remained consistent, confirming the stability and reliability of the pooled results (see Supplementary Materials for details).

For the primary outcome measure PWT, which exhibited substantial heterogeneity, we conducted further investigations to identify potential sources through subgroup analysis and meta-regression. Studies were categorized into eight subgroups based on the following factors: model type, animal species, sample size, acupoint location, stimulation frequency, treatment duration, treatment period, and quality assessment score. The pooled analysis indicated that none of these factors significantly contributed to the observed heterogeneity. Detailed results of the subgroup analyses are provided in the Supplementary Materials. The findings of the meta-regression are summarized in Table 4.

**Table 4:** Meta-regression results for the paw withdrawal threshold (PWT) outcome.

Outcomes	Variable	P value
PWT	Model	0.966
	Animal	0.530
	Number	0.914
	Acupuncture location	0.360
	Frequency	0.561
	Time	0.932
	Cot	0.879
	Scores	0.750

### Assessment of publication bias

Publication bias assessment for the 12 studies reporting PWT indicated funnel plot asymmetry (*t=*5.22, P<0.001), suggesting potential publication bias. Further application of a contour-enhanced funnel plot and trim-and-fill analysis to the PWT data confirmed the presence of significant publication bias (P<0.001) (Figure 3).

**Figure 3:**
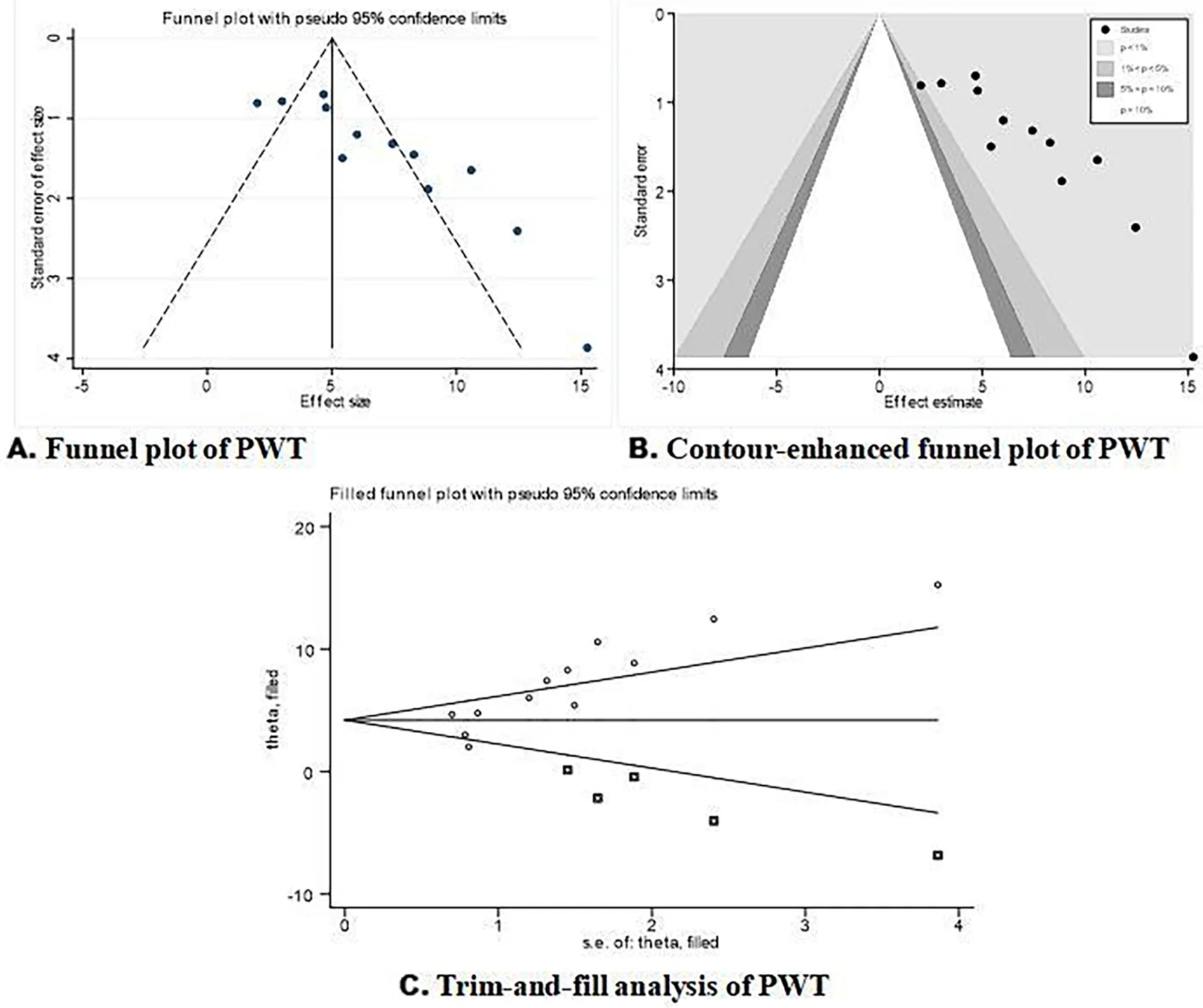
Analysis of publication bias for paw withdrawal threshold (PWT).

## Discussion

### EA exerted multi-target regulatory effects on CIPN

This systematic review and meta-analysis synthesized current preclinical evidence on EA for the treatment of CIPN-related pain, comprehensively evaluating its multidimensional effects, including improvements in behavioral symptoms, recovery of physiological function, and modulation of molecular pathological pathways. The integrated analysis demonstrated that EA intervention significantly alleviated pain-related behaviors and restored nerve conduction function in CIPN animal models. Its therapeutic mechanism involved multi-target regulation of several interconnected pathological processes, including neuroinflammation, oxidative stress, and peripheral neuronal sensitization. These findings provide a scientific biological basis for EA as a potential non-pharmacological adjuvant therapy and offer robust experimental evidence elucidating its analgesic and neuroprotective mechanisms.

#### Behavioral phenotype and physiological state

The core clinical manifestations of CIPN encompass a spectrum of sensory abnormalities, with mechanical allodynia, thermal hyperalgesia, and cold allodynia being the most prevalent, directly impacting patient quality of life (27). In animal models, a decreased mechanical pain threshold reflects pain evoked by light touch, a reduced thermal pain threshold corresponds to increased sensitivity to heat stimuli, and cold allodynia manifests as a painful response to non-noxious cold stimulation (28). Behavioral pain assessment serves as a direct window into CIPN-related pain. This study found that EA significantly increased the mechanical and thermal pain thresholds and alleviated cold allodynia in CIPN animals. This suggests that EA intervention directly targets the peripheral and central sensitization processes underlying pain perception, effectively mitigating the three most characteristic pain hypersensitivities in CIPN. EA is capable of directly modulating pain-related regions in both the central and peripheral nervous systems (29). This comprehensive behavioral improvement strongly indicated that EA does not merely target a single pain modality but likely exerts broad-spectrum analgesic effects and reverses pain hypersensitivity by regulating common upstream mechanisms of pain generation. This provides a reliable phenotypic foundation for subsequent exploration of molecular mechanisms.

At the physiological level, the body weight gain rate serves as a sensitive indicator of overall animal health and systemic toxicity burden from chemotherapy, while NCV directly quantifies the functional integrity of peripheral nerves. A reduction in NCV objectively reflects axonal pathology in CIPN (30). These parameters are often compromised in CIPN due to neural damage and systemic distress (31). This study found that EA significantly improved NCV, but its effect on increasing the weight gain rate did not reach statistical significance. This suggests that the therapeutic benefits of EA may be more preferentially and specifically directed at the pathological processes within the nervous system, rather than broadly reversing the systemic metabolic consumption induced by chemotherapy. Body weight, as a macro-level indicator influenced by multiple factors, may require more comprehensive intervention strategies for improvement. The improvement in NCV holds significance not only for functional recovery but also, more fundamentally, for establishing a structural basis for pain alleviation. It signifies protection of myelin and axonal structures, which helps reduce abnormal spontaneous discharges in sensory neurons (32). Furthermore, this improvement primarily reflects the recovery of conduction function in myelinated sensory fibers (particularly Aβ fibers), thereby potentially enhancing endogenous gating inhibition of pain signals at the spinal dorsal horn (33). This indicates that through its distinct neuroprotective effects, EA not only preserves physiological electrical signal conduction along axons but also provides a key electrophysiological mechanism explaining the downstream improvement in pain behaviors, collectively substantiating the substantial benefit of EA in treating CIPN-related pain (34).

#### Neuroinflammation

Neuroinflammation is a central pathological mechanism driving the onset and maintenance of CIPN (35). Chemotherapeutic agents induce injury to the dorsal root ganglion and sciatic nerve, leading to the release of endogenous danger signals. These signals activate pattern recognition receptors such as TLR4, thereby initiating downstream inflammatory signaling cascades. The activation of TLR4 not only initiates the inflammatory response but can also directly modulate the expression of pain-related genes. Activated TLR4 triggers NF-κB. As a key transcriptional hub for both pain and inflammation, the activation of NF-κB drives the robust expression of downstream effector molecules, including TNF-α and IL-1β, which exacerbate neuropathic pain (36,37). These pro-inflammatory cytokines are potent mediators of pain sensitization. They can directly enhance the excitability of nociceptive neurons and further activate and sustain the reactive state of glial cells (38). The activation of astrocytes, marked by the upregulated expression of GFAP, plays a significant role in central sensitization by releasing additional inflammatory mediators, creating a self-perpetuating cycle that drives persistent pain (39). The results of this meta-analysis demonstrated that EA treatment significantly downregulated the levels of TLR4, TNF-α, IL-1β, and GFAP. This coordinated suppression delineates a chain of clear evidence indicating that EA inhibits the complete neuroinflammatory pathway encompassing the TLR4/NF-κB signaling axis, pro-inflammatory cytokine release, and astrocyte activation. By disrupting the initiation and amplification stages of the inflammatory response, EA fosters an anti-inflammatory microenvironment for the damaged neural tissue. More directly, it intervenes in the processes of pain signal amplification and central integration driven by this inflammatory network, representing a key mechanism through which EA alleviates neuropathic pain.

#### Oxidative stress damage

Oxidative stress is intricately linked with neuroinflammation, together constituting early injurious events in CIPN (40). Chemotherapeutic agents directly generate substantial reactive oxygen species during metabolism, while inflammatory processes can further exacerbate the oxidative burden (41). Excessive oxidative stress leads to lipid, protein, and DNA damage. These oxidative products can directly activate nociceptors, such as TRPA1 and TRPV1, thereby translating oxidative damage into pain signals (42,43). Nrf2 is a master transcriptional regulator of the cellular antioxidant defense system. Activated under oxidative stress, Nrf2 initiates the expression of a battery of phase II detoxifying enzymes and antioxidant proteins, facilitating the clearance of free radicals and mitigating oxidative damage. This represents a crucial endogenous protective mechanism against cellular injury (44,45). In the context of CIPN, this defense system is often functionally impaired. The present study found that EA treatment significantly upregulated the protein expression level of Nrf2 in the dorsal root ganglia of CIPN animal models. By activating the Nrf2 pathway, EA enhances tissue antioxidant capacity, protecting the structure and function of neurons and Schwann cells (46,47). This intervention acts at the source of pain generation. On one hand, it clears oxidative products capable of directly activating TRP channels; on the other, it disrupts the vicious cycle between oxidation and inflammation, ultimately alleviating neuronal sensitization. This mechanism engages in crosstalk with the suppression of the NF-κB inflammatory pathway. Their synergistic interaction forms a dual foundation for the neuroprotective and analgesic effects of EA.

#### Peripheral neuronal sensitization

Neuronal sensitization, particularly the heightened excitability of peripheral nociceptors, represents a final common pathway in the generation of CIPN pain symptoms (48). Transient receptor potential (TRP) channels, notably TRPA1 and TRPV1, play a pivotal role as “molecular sensors” in this process. TRPV1, the predominant thermal nociceptor, contributes directly to heat-evoked pain when its activity is enhanced (49). TRPA1, a polymodal sensor, is critically involved in mechanical and cold allodynia and is highly sensitive to oxidative stress products (50,51). Upstream pathological events, such as the release of pro-inflammatory cytokines (TNF-α, IL-1β) and the accumulation of reactive oxygen species, can directly phosphorylate and sensitize these channels or upregulate their expression. This leads to neuronal hyper-responsiveness to normal or sub-threshold stimuli, manifesting as pain hypersensitivity (52). The results of this study show that EA intervention significantly reduced the expression levels of TRPA1 and TRPV1 in the dorsal root ganglia or sciatic nerve. This effect is likely a plausible downstream consequence of EA's successful suppression of upstream neuroinflammation and oxidative stress. By precisely downregulating the expression and function of these terminal effectors in pain signal transduction, EA effectively attenuates the intensity of nociceptive input to the central nervous system. This provides a unified and specific cellular-molecular explanation for the observed improvement across multiple pain modalities (mechanical, thermal, cold) at the behavioral level.

### Comparison with existing research

Compared with previous reviews that primarily focused on the clinical efficacy of EA or isolated molecular targets, this study, by concentrating on rigorous animal experiments and conducting a meta-analysis, is the first to systematically integrate a multi-dimensional chain of evidence at the mechanistic level for EA in alleviating CIPN pain. In contrast to research mainly concerned with changes in clinical pain scores, this study not only confirmed the overall effect of EA on improving mechanical, thermal, and cold hypersensitivity in behavioral terms but, more importantly, revealed the intrinsic logical connection between its “neuroprotective-analgesic” actions by synthesizing neurophysiological and molecular biological indicators. This analysis addressed the common limitations of prior studies, which often remained at the level of phenomenological description or isolated examination of single pathways. It constructed a coherent pathophysiological model spanning from upstream injury modulation to downstream signal transduction, thereby providing more robust and systematic experimental evidence for the biological plausibility of EA in treating CIPN-related pain.

### Limitations

This systematic review and meta-analysis included 13 randomized controlled trials identified through a comprehensive literature search. It should be noted that all included studies originated from Asian countries, which reflects the research activity in this region regarding EA but also indicates a potential geographical limitation of the current evidence. Consequently, caution is warranted when generalizing the findings to other regions, such as Europe. Future multinational, collaborative studies involving diverse cultural and linguistic contexts are necessary to further validate the generalizability and reproducibility of EA's effects. Significant heterogeneity (*l* ^2^>50%) was observed across several outcome measures, including PWT, PWL, cold allodynia, weight gain, TNF-α, IL-1β, TRPA1, and TRPV1. For the primary outcome PWT, funnel plot asymmetry suggested potential publication bias, which was confirmed by contour-enhanced funnel plot and trim-and-fill analyses. This publication bias may represent a key source of heterogeneity for PWT and could lead to an overestimation of EA's true treatment effect. Although sensitivity analyses were performed to assess the robustness of the results, these statistical methods can only partially adjust for and evaluate such bias but cannot eliminate it entirely. Furthermore, subgroup analysis and meta-regression examining eight potential moderators failed to identify significant sources of the remaining heterogeneity. This highlights the need for improved methodological rigor and reporting transparency in future studies. The heterogeneity observed for other outcomes may be attributable to variations in treatment protocols, such as electrical stimulation parameters (current, frequency), acupoint selection and combination, treatment duration and frequency, and specific manipulation techniques. These elements require standardization and detailed reporting in subsequent research.

A systematic assessment of the methodological quality and risk of bias of the included studies revealed several key limitations that may affect the reliability of the conclusions: 1) Inadequate reporting of randomization procedures. Only three studies explicitly described the method of allocation concealment, and only two detailed the generation of the random sequence. Incomplete reporting, even if randomization was performed, significantly increases the risk of selection bias and undermines the credibility of the findings. Future studies must provide complete and transparent reporting of the entire randomization process; 2) Insufficient implementation and reporting of blinding. Only four studies mentioned blinding of outcome assessors, and none reported whether blinding was applied during modeling or intervention procedures. The absence of blinding may introduce performance and detection bias, compromising the objectivity of the results; 3) Incomplete reporting of conflicts of interest. Seven studies did not include a statement regarding conflicts of interest, reflecting a general shortcoming in reporting standards within this field. We strongly recommend that future research adhere strictly to guidelines for systematic reviews of animal studies, such as those from SYRCLE, to enhance transparency and credibility; 4) Lack of reported sample size calculations. None of the studies described the basis or process for determining sample size, which undermines the reproducibility and scientific rigor of the experimental design. Adequate sample size estimation is fundamental to ensuring statistical power and should be standardly reported; 5) Need for standardized reporting of animal welfare and ethics. Seven studies did not clearly describe measures related to animal welfare. While we assume these studies complied with ethical standards, clear reporting is essential not only for ethical review but also for enhancing the completeness and reproducibility of the experimental process.

### Clinical significance

The multi-target mechanisms of action of EA integrated in this systematic review offer a novel strategic perspective for the clinical management of the challenging symptom of CIPN-related pain. Current pharmacological options for CIPN pain are limited and often associated with side effects, frequently constrained by insufficient efficacy or adverse reactions. This study indicated that EA may offer an integrated analgesic strategy. It not only mitigates neuropathic injury through anti-inflammatory and antioxidant mechanisms but also directly modulates key ion channels in pain signal transduction, such as TRPV1 and TRPA1, thereby synergistically alleviating pain across multiple pathological stages. This suggests that in clinical practice, EA could potentially serve as an early intervention to prevent the emergence of pain hypersensitivity or block its chronification.

## Conclusions and Prospects

Based on this systematic review and meta-analysis, current animal experimental evidence indicated that EA can effectively alleviate pain symptoms associated with CIPN through a multi-target synergistic regulatory mechanism. At the behavioral level, EA significantly increased mechanical and thermal pain thresholds and alleviated cold allodynia. Regarding physiological function, EA effectively improved nerve conduction velocity. At the molecular level, EA upregulated the expression of the core antioxidant factor Nrf2, while simultaneously suppressing the TLR4/NF-κB inflammatory signaling axis and its downstream effectors - TNF-α, IL-1β, and GFAP - and reduced the expression of the nociceptors TRPV1 and TRPA1.

In summary, EA operated via an integrated mechanism of “anti-inflammation, anti-oxidation, and neuronal desensitization”. This provides systematic preclinical evidence supporting its potential clinical use as a multi-target adjuvant strategy for analgesia, neuroprotection, and functional restoration. Future mechanism-driven, high-quality clinical studies are warranted to validate and optimize personalized EA treatment protocols for patients.

## Data Availability

The datasets generated and analyzed in this meta-analysis, including extracted data and analytical codes, are accessible via the following databases: PubMed, Web of Science (WOS), Embase, MEDLINE, the Cochrane Library, Scopus, China National Knowledge Infrastructure (CNKI), SinoMed, WanFang Data, and VIP Database. The original studies are cited in the reference list. Detailed datasets and analysis codes have been included in the Supplementary Materials.

## Supplementary Materials

Click to view [pdf].
