# Supplementary material for: Experimental evidence for electroacupuncture in managing pain from chemotherapy-induced peripheral neuropathy: a systematic review and meta-analysis
Source: Braz J Med Biol Res. 2026 Oct 30;59:e15562. doi: 10.1590/1414-431X2026e15562 (PMC13722001; doi:10.1590/1414-431X2026e15562)
Supplement: Supplementary file 1 [file 1414-431X-bjmbr-59-e15562-suppl.pdf]

Supplementary Material

A
Search Query

Pubmed
(((((Acupuncture[Title/Abstract] OR Electroacupuncture[Title/Abstract] OR Electric acupuncture[Title/Abstract] OR Electro-acupuncture[Title/Abstract] OR Acupoint[Title/Abstract]) OR (Acupuncture or Electroacupuncture or Electric acupuncture or Electro-acupuncture or Acupoint[MeSH Terms])) AND ((Cancer[Title/Abstract] OR Neoplasia[Title/Abstract] OR Neoplasm[Title/Abstract] OR Tumor[Title/Abstract] OR Malignant Neoplasm[Title/Abstract] OR Malignancy[Title/Abstract] OR Malignancies[Title/Abstract] OR Malignant Neoplasms[Title/Abstract] OR Neoplasm, Malignant[Title/Abstract] OR Chemotherapy[Title/Abstract] OR Chemotherapies[Title/Abstract] OR Pharmacotherapy[Title/Abstract] OR Pharmacotherapies[Title/Abstract] OR Therapy, Drug[Title/Abstract] OR Drug Therapies[Title/Abstract] OR Therapies, Drug[Title/Abstract]) OR (Cancer or Neoplasia or Neoplasm or Tumor or Malignant Neoplasm or Malignancy or Malignancies or Malignant Neoplasms or Neoplasm, Malignant or Chemotherapy or Chemotherapies or Pharmacotherapy or Pharmacotherapies or Therapy, Drug or Drug Therapies or Therapies, Drug[MeSH Terms]))) AND ((peripheral neuropathy[Title/Abstract] OR Neurotoxicity[Title/Abstract] OR Nervous System Poisonings[Title/Abstract] OR Peripheral nervous system diseases[Title/Abstract] OR PNS Disease[Title/Abstract]) OR (peripheral neuropathy or Neurotoxicity or Nervous System Poisonings or Peripheral nervous system diseases or PNS Disease[MeSH Terms]))) AND ((Rat[Title/Abstract] OR Mice[Title/Abstract] OR Mouse[Title/Abstract] OR animal[Title/Abstract]) OR (Rat or Mice or Mouse or animal[MeSH Terms]))) NOT ((Meta analysis[Title/Abstract] OR meta-analyses[Title/Abstract] OR Meta-Analysis[Title/Abstract] OR Network Meta-Analysis[Title/Abstract] OR Case reports[Title/Abstract] OR Case Histories[Title/Abstract] OR Case Study[Title/Abstract] OR Case Studies[Title/Abstract] OR cross-over studies[Title/Abstract] OR Cross-Over Study[Title/Abstract] OR Studies, Cross-Over[Title/Abstract] OR Study, Cross-Over[Title/Abstract] OR Crossover Trials[Title/Abstract] OR Trial, Crossover[Title/Abstract] OR Cross-Over Trials[Title/Abstract] OR Cross Over Trials[Title/Abstract] OR Trial, Cross-Over[Title/Abstract] OR Crossover Studies[Title/Abstract] OR Crossover Study[Title/Abstract] OR Studies, Crossover[Title/Abstract] OR Study, Crossover[Title/Abstract] OR Cross-Over Design[Title/Abstract] OR Design, Cross-Over[Title/Abstract] OR Crossover Design[Title/Abstract] OR Design, Crossover[Title/Abstract] OR reviews[Title/Abstract] OR State-of-the-Art Review[Title/Abstract] OR State of the Art Review[Title/Abstract] OR State-of-the-Art Reviews[Title/Abstract] OR Review, State-of-the-Art[Title/Abstract] OR State of the Art Review[Title/Abstract] OR Systematic review[Title/Abstract] OR meeting abstracts[Title/Abstract] OR Meeting Abstracts[Title/Abstract] OR Congress[Title/Abstract] OR RCT[Title/Abstract] OR randomized controlled trial[Title/Abstract]) OR (Meta analysis or meta-analyses or Meta-Analysis or Network Meta-Analysis or Case reports or Case Histories or

Case Study or Case Studies or cross-over studies or Cross-Over Study or Studies, Cross-Over or Study, Cross-Over or Crossover Trials or Trial, Crossover or Cross-Over Trials or Cross Over Trials or Trial, Cross-Over or Crossover Studies or Crossover Study or Studies, Crossover or Study, Crossover or Cross-Over Design or Design, Cross-Over or Crossover Design or Design, Crossover or reviews or State-of-the-Art Review or State of the Art Review or State-of-the-Art Reviews or Review, State-of-the-Art or State of the Art Review or Systematic review or meeting abstracts or Meeting Abstracts or Congress or RCT or randomized controlled trial[MeSH Terms]))

Wos

Acupuncture or Electroacupuncture or Electric acupuncture or Electro-acupuncture or Acupoint (Topic) and Cancer or Neoplasia or Neoplasm or Tumor or Malignant Neoplasm or Malignancy or Malignancies or Malignant Neoplasms or Neoplasm, Malignant or Chemotherapy or Chemotherapies or Pharmacotherapy or Pharmacotherapies or Therapy, Drug or Drug Therapies or Therapies, Drug (Topic) and peripheral neuropathy or Neurotoxicity or Nervous System Poisonings or Peripheral nervous system diseases or PNS Disease (Topic) and Rat or Mice or Mouse or animal (Topic) not Meta analysis or meta-analyses or Meta-Analysis or Network Meta-Analysis or Case reports or Case Histories or Case Study or Case Studies or cross-over studies or Cross-Over Study or Studies, Cross-Over or Study, Cross-Over or Crossover Trials or Trial, Crossover or Cross-Over Trials or Cross Over Trials or Trial, Cross-Over or Crossover Studies or Crossover Study or Studies, Crossover or Study, Crossover or Cross-Over Design or Design, Cross-Over or Crossover Design or Design, Crossover or reviews or State-of-the-Art Review or State of the Art Review or State-of-the-Art Reviews or Review, State-of-the-Art or State of the Art Review or Systematic review or meeting abstracts or Meeting Abstracts or Congress or RCT or randomized controlled trial (Topic)

Embase

(acupuncture:ti,ab,kw OR electroacupuncture:ti,ab,kw OR 'electric acupuncture':ti,ab,kw OR 'electro acupuncture':ti,ab,kw OR 'acupuncture point':ti,ab,kw) AND (cancer:ti,ab,kw OR neoplasia:ti,ab,kw OR neoplasm:ti,ab,kw OR tumor:ti,ab,kw OR 'malignant neoplasm':ti,ab,kw OR malignancy:ti,ab,kw OR malignancies:ti,ab,kw OR 'malignant neoplasms':ti,ab,kw OR 'neoplasm, malignant':ti,ab,kw OR chemotherapy:ti,ab,kw OR chemotherapies:ti,ab,kw OR pharmacotherapy:ti,ab,kw OR pharmacotherapies:ti,ab,kw OR 'therapy, drug':ti,ab,kw OR 'drug therapies':ti,ab,kw OR 'therapies, drug':ti,ab,kw) AND ('peripheral neuropathy':ti,ab,kw OR neurotoxicity:ti,ab,kw OR 'nervous system poisonings':ti,ab,kw OR 'peripheral nervous system diseases':ti,ab,kw OR 'pns disease':ti,ab,kw) AND (rat:ti,ab,kw OR mice:ti,ab,kw OR mouse:ti,ab,kw OR 'animal':ti,ab,kw) NOT ('meta analyses':ti,ab,kw OR 'meta analysis':ti,ab,kw OR 'network meta-analysis':ti,ab,kw OR 'case reports':ti,ab,kw OR 'case histories':ti,ab,kw OR 'case study':ti,ab,kw OR 'case studies':ti,ab,kw OR 'cross-over studies':ti,ab,kw OR 'cross-over study':ti,ab,kw OR 'studies, cross-over':ti,ab,kw OR 'study, cross-over':ti,ab,kw OR 'crossover trials':ti,ab,kw OR 'trial,

crossover':ti,ab,kw OR 'cross-over trials':ti,ab,kw OR 'cross over trials':ti,ab,kw OR 'trial, cross-over':ti,ab,kw OR 'crossover studies':ti,ab,kw OR 'crossover study':ti,ab,kw OR 'studies, crossover':ti,ab,kw OR 'study, crossover':ti,ab,kw OR 'cross-over design':ti,ab,kw OR 'design, cross-over':ti,ab,kw OR 'crossover design':ti,ab,kw OR 'design, crossover':ti,ab,kw OR reviews:ti,ab,kw OR 'state-of-the-art review':ti,ab,kw OR 'state-of-the-art reviews':ti,ab,kw OR 'review, state-of-the-art':ti,ab,kw OR 'state of the art review':ti,ab,kw OR 'systematic review':ti,ab,kw OR 'meeting abstracts':ti,ab,kw OR congress:ti,ab,kw OR rct:ti,ab,kw OR 'randomized controlled trial':ti,ab,kw)

Scopus
(TITLE-ABS-KEY (Acupuncture OR Electroacupuncture OR Electric acupuncture OR Electro-acupuncture OR Acupoint) AND TITLE-ABS-KEY (Cancer OR Neoplasia OR Neoplasm OR Tumor OR Malignant Neoplasm OR Malignancy OR Malignancies OR Malignant Neoplasms OR Neoplasm , Malignant OR Chemotherapy OR Chemotherapies OR Pharmacotherapy OR Pharmacotherapies OR Therapy , Drug OR Drug Therapies OR Therapies , Drug) AND TITLE-ABS-KEY (peripheral neuropathy OR Neurotoxicity OR Nervous System Poisonings OR Peripheral nervous system diseases OR PNS Disease) AND TITLE-ABS-KEY (Rat OR Mice OR Mouse OR animal) AND NOT TITLE-ABS-KEY (Meta analysis OR meta-analyses OR Meta-Analysis OR Network Meta-Analysis OR Case reports OR Case Histories OR Case Study OR Case Studies OR cross-over studies OR Cross-Over Study OR Studies , Cross-Over OR Study , Cross-Over OR Crossover Trials OR Trial , Crossover OR Cross-Over Trials OR Cross Over Trials OR Trial , Cross-Over OR Crossover Studies OR Crossover Study OR Studies , Crossover OR Study , Crossover OR Cross-Over Design OR Design , Cross-Over OR Crossover Design OR Design , Crossover OR reviews OR State-of-the-Art Review OR State of the Art Review OR State-of-the-Art Reviews OR Review , State-of-the-Art OR State of the Art Review OR Systematic review OR meeting abstracts OR Meeting Abstracts OR Congress OR RCT OR randomized controlled trial))

Medline
XB (Acupuncture or Electroacupuncture or Electric acupuncture or Electro-acupuncture or Acupoint) AND XB (Cancer or Neoplasia or Neoplasm or Tumor or Malignant Neoplasm or Malignancy or Malignancies or Malignant Neoplasms or Neoplasm, Malignant or Chemotherapy or Chemotherapies or Pharmacotherapy or Pharmacotherapies or Therapy, Drug or Drug Therapies or Therapies, Drug) AND XB (peripheral neuropathy or Neurotoxicity or Nervous System Poisonings or Peripheral nervous system diseases or PNS Disease) AND XB (Rat or Mice or Mouse or animal) NOT XB (Meta analysis or meta-analyses or Meta-Analysis or Network Meta-Analysis or Case reports or Case Histories or Case Study or Case Studies or cross-over studies or Cross-Over Study or Studies, Cross-Over or Study, Cross-Over or Crossover Trials or Trial, Crossover or Cross-Over Trials or Cross Over Trials or Trial, Cross-Over or Crossover Studies or Crossover Study or Studies, Crossover or Study, Crossover or Cross-Over Design or Design, Cross-

Over or Crossover Design or Design, Crossover or reviews or State-of-the-Art Review or State of the Art Review or State-of-the-Art Reviews or Review, State-of-the-Art or State of the Art Review or Systematic review or meeting abstracts or Meeting Abstracts or Congress or RCT or randomized controlled trial)

Cochrane library
Acupuncture or Electroacupuncture or Electric acupuncture or Electro-acupuncture or Acupoint in Title Abstract Keyword AND Cancer or Neoplasia or Neoplasm or Tumor or Malignant Neoplasm or Malignancy or Malignancies or Malignant Neoplasms or Neoplasm, Malignant or Chemotherapy or Chemotherapies or Pharmacotherapy or Pharmacotherapies or Therapy, Drug or Drug Therapies or Therapies, Drug in Title Abstract Keyword AND peripheral neuropathy or Neurotoxicity or Nervous System Poisonings or Peripheral nervous system diseases or PNS Disease in Title Abstract Keyword AND Rat or Mice or Mouse or animal in Title Abstract Keyword NOT Meta analysis or meta-analyses or Meta-Analysis or Network Meta-Analysis or Case reports or Case Histories or Case Study or Case Studies or cross-over studies or Cross-Over Study or Studies, Cross-Over or Study, Cross-Over or Crossover Trials or Trial, Crossover or Cross-Over Trials or Cross Over Trials or Trial, Cross-Over or Crossover Studies or Crossover Study or Studies, Crossover or Study, Crossover or Cross-Over Design or Design, Cross-Over or Crossover Design or Design, Crossover or reviews or State-of-the-Art Review or State of the Art Review or State-of-the-Art Reviews or Review, State-of-the-Art or State of the Art Review or Systematic review or meeting abstracts or Meeting Abstracts or Congress or RCT or randomized controlled trial in Record Title - (Word variations have been searched)

CNKI
（篇关摘：针刺 + 电针 + 穴位(精确)）AND（篇关摘：癌症 + 肿瘤 + 化疗(精确)）AND（篇关摘：周围神经损伤 + 周围神经病变 + 神经毒性 + 周围神经系统疾病(精确)）AND（篇关摘：鼠 + 动物(精确)）NOT（篇关摘：meta 分析 + 病例报告 + 案例分析 + 交叉试验 + 会议 + 研究进展 + 指南 + 文献研究 + 理论(精确)）

SinoMed
("针刺"[常用字段:智能] OR "电针"[常用字段:智能] OR "穴位"[常用字段:智能]) AND("癌症"[常用字段:智能] OR "肿瘤"[常用字段:智能] OR "化疗"[常用字段:智能]) AND("周围神经损伤"[常用字段:智能] OR "周围神经病变"[常用字段:智能] OR "神经毒性"[常用字段:智能] OR "周围神经系统疾病"[常用字段:智能]) AND("鼠"[常用字段:智能] OR "动物"[常用字段:智能]) NOT("meta 分析"[常用字段:智能] OR "病例报告"[常用字段:智能] OR "案例分析"[常用字段:智能] OR "交叉试验"[常用字段:智能] OR "会议"[常用字段:智能] OR "研究进展"[常用字段:智能] OR "指南"[常用字段:智能] OR "文献研究"[常用字段:智能] OR "理论"[常用字段:智能])

WangFang

检索表达式（中英文扩展&主题词扩展）： 全部:(针刺 OR 电针 OR 穴位) and 全部:(癌症 OR 肿瘤 OR 化疗) and 全部:(周围神经损伤 OR 周围神经病变 OR 神经毒性 OR 周围神经系统疾病) and 全部:(鼠 OR 动物) not 题名或关键词:(meta 分析 OR 病例报告 OR 案例分析 OR 交叉试验 OR 会议 OR 研究进展 OR 指南 OR 文献研究 OR 理论)

VIP

((((((任意字段=针刺 OR 任意字段=电针) OR 任意字段=穴位) AND ((任意字段=癌症 OR 任意字段=肿瘤) OR 任意字段=化疗)) AND (((任意字段=周围神经损伤 OR 任意字段=周围神经病变) OR 任意字段=神经毒性) OR 任意字段=周围神经系统疾病)) AND (任意字段=鼠 OR 任意字段=动物)) AND (NOT ((((((((任意字段=meta 分析 OR 任意字段=病例报告) OR 任意字段=案例分析) OR 任意字段=交叉试验) OR 任意字段=会议) OR 任意字段=研究进展) OR 任意字段=指南) OR 任意字段=文献研究) OR 任意字段=理论)))

B

SYRCLE's Risk of Bias (ROB) Tool

The 10 items were assessed individually, with the ten domains classified as low, unclear, or high risk of bias including sequence generation, baseline characteristics, allocation concealment, random housing, blinding of participants/personnel, random outcome assessment, blinding of the assessor, incomplete outcome data, selective outcome reporting, and other bias. Blinding of participants/personnel was deemed “not applicable” given the impossibility of adequate blinding for acupuncture practitioners.

(1) Generation of animal allocation sequence was random; (2) each group was similar or was at adjusted at baseline; (3) the allocation was adequately concealed; (4) animals were housed at random; (5) both animal breeders and researchers were blinded for the intervention of each animal received; (6) animals were selected randomly for outcome evaluation; (7) outcome evaluator was blinded; (8) the incomplete outcome data were absolutely addressed; (9) reports of the research were free of selective outcome reporting; (10) study was evidently free of other potential issues which may cause bias.

CAMARADES checklist

The 10 items were counted separately:1) peer-reviewed journal; 2) temperature control; 3) animals were randomly allocated; 4) blind established model; 5) blinded outcome assessment; 6) reporting of animals excluded from analysis; 7)

appropriate animal model; 8) calculation of sample size; 9) statement of compliance with animal welfare regulations; 10) possible conflicts of interest. One point was assigned to each of the ten items on the scale.

C
Sensitivity Analyses

[Yuanyuan Li 2019(a): reference 18; Yuanyuan Li 2019(b): reference 23]

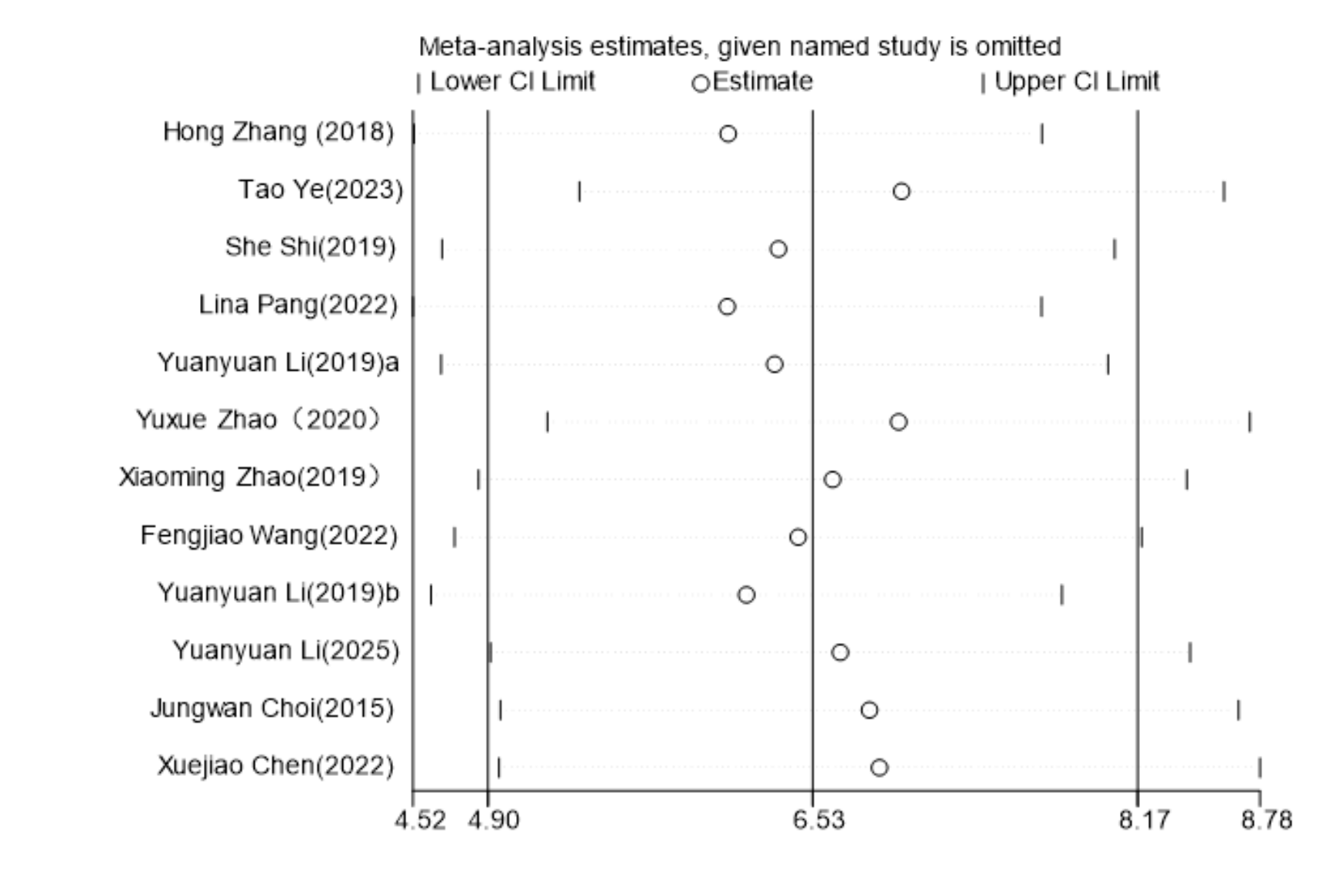

Paw withdrawal threshold (PWT) - Sensitivity Analyses

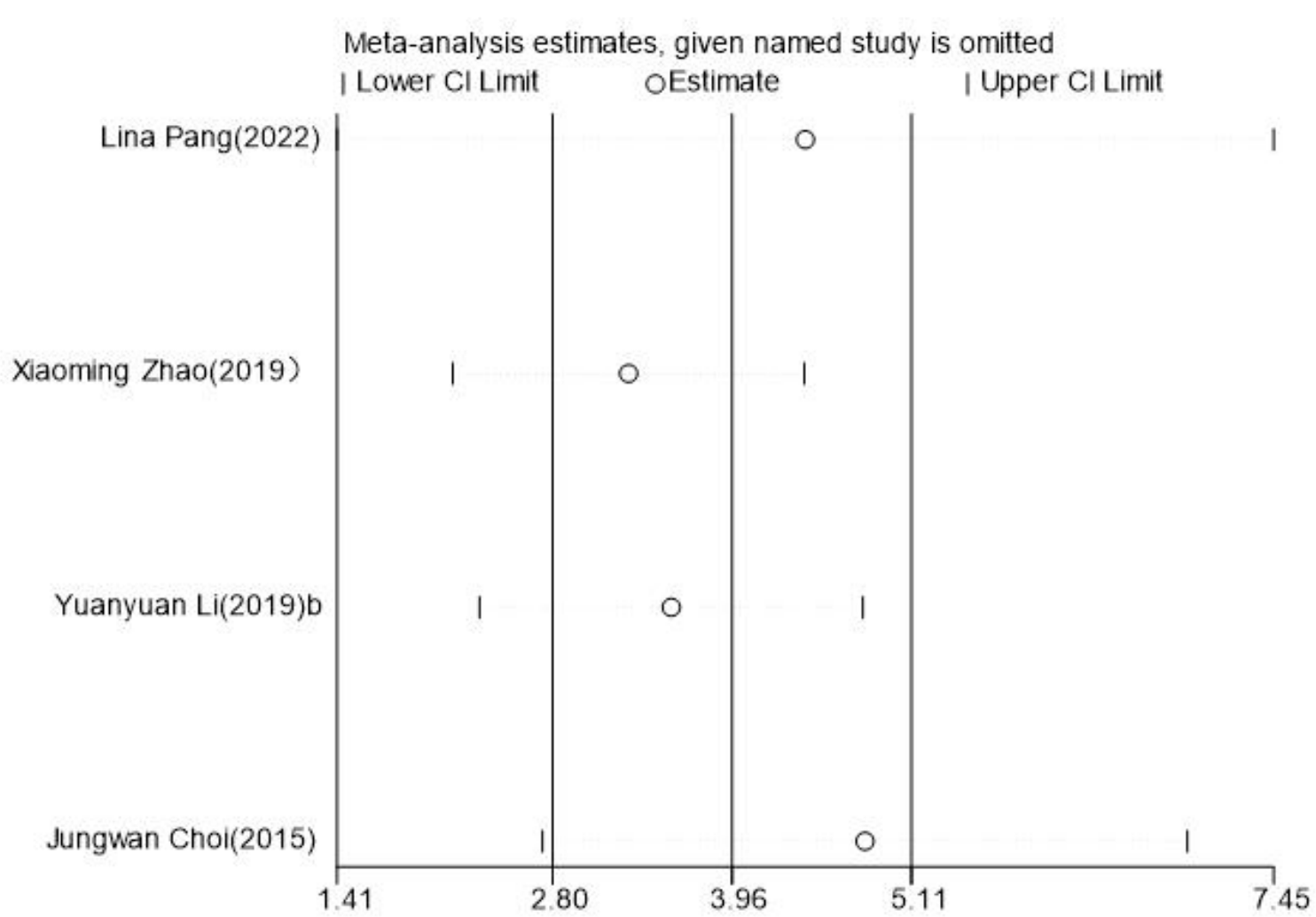

Pain withdrawal latency (PWL) - Sensitivity Analyses

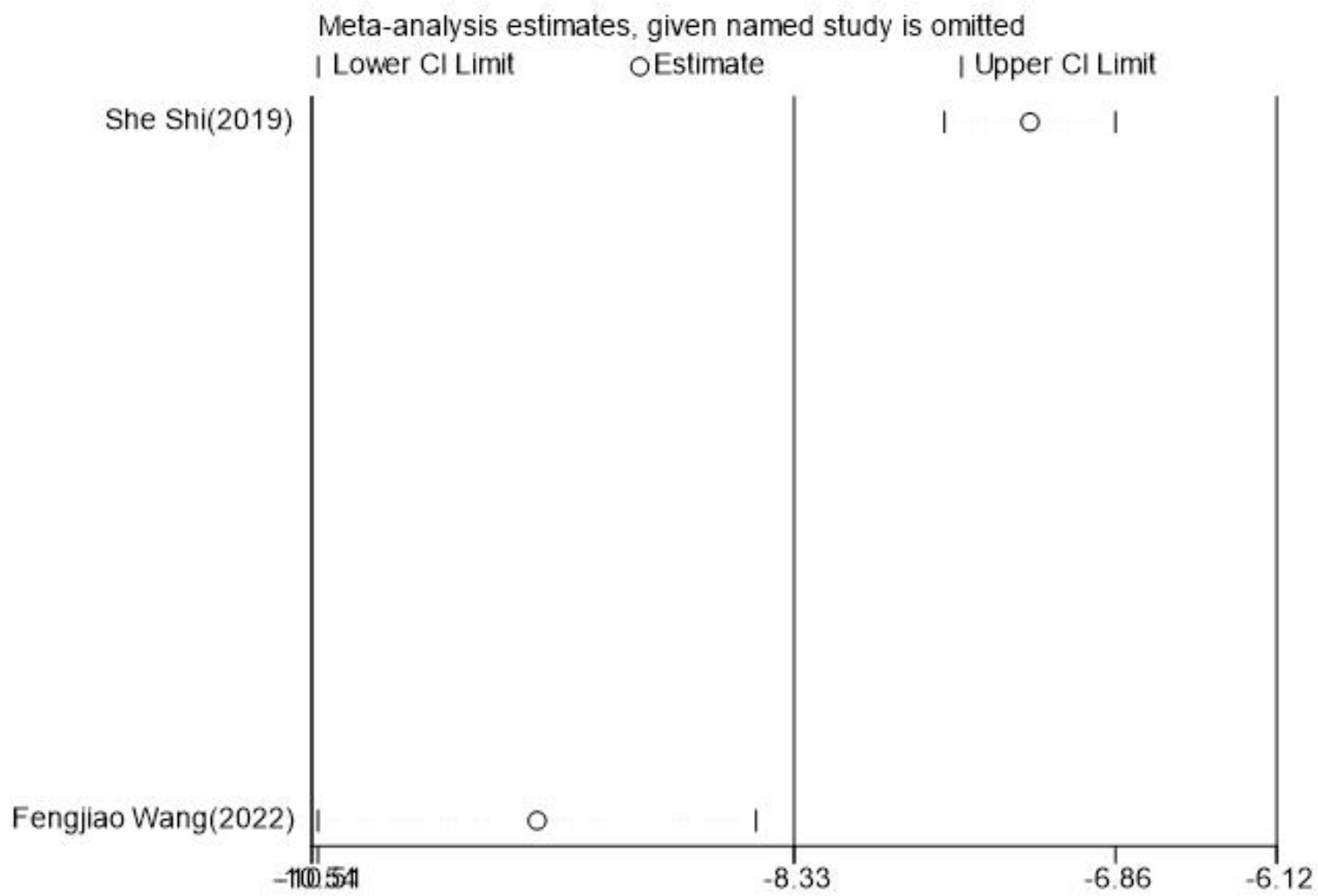

Cold allodynia - Sensitivity Analyses

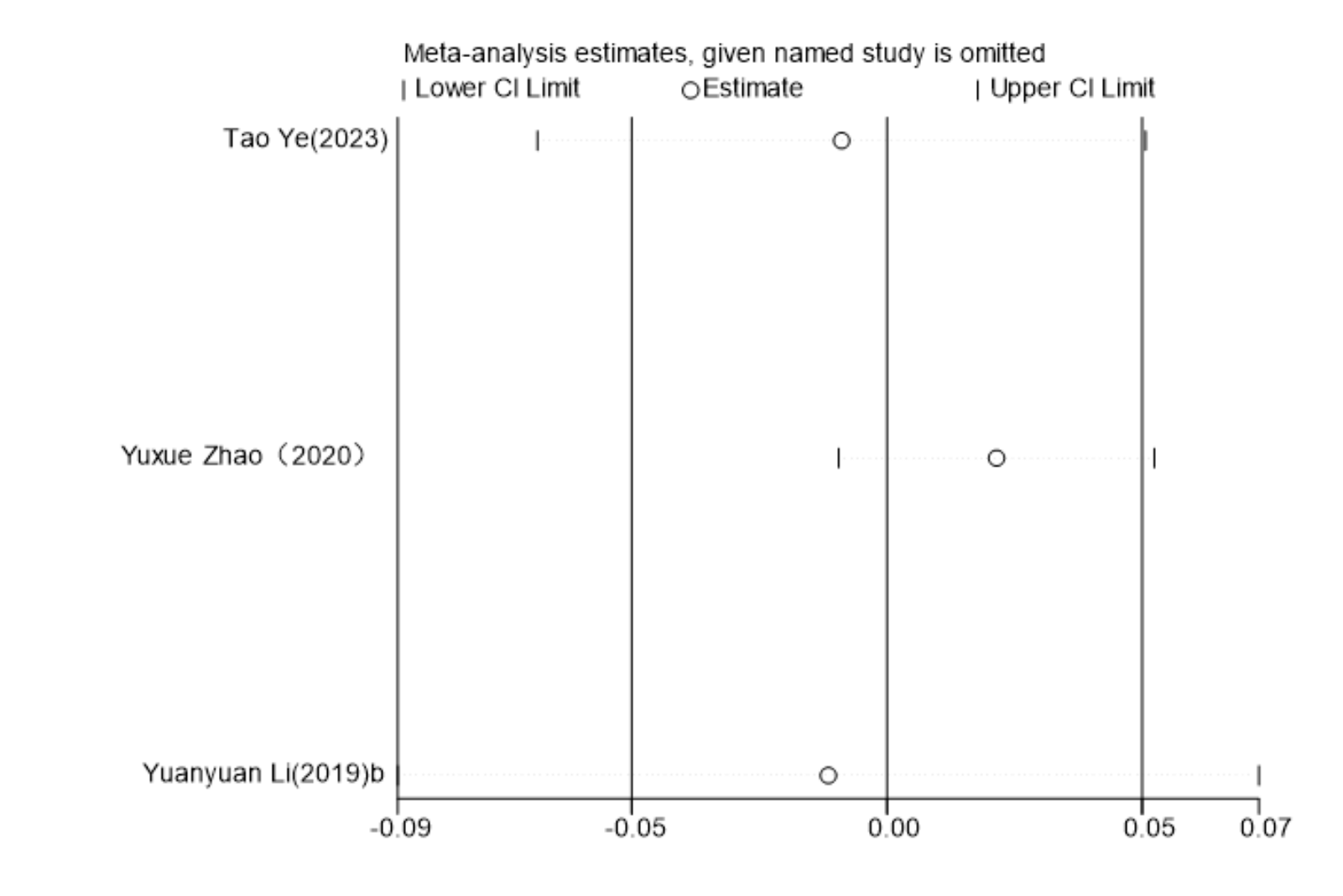

Weight gain - Sensitivity Analyses

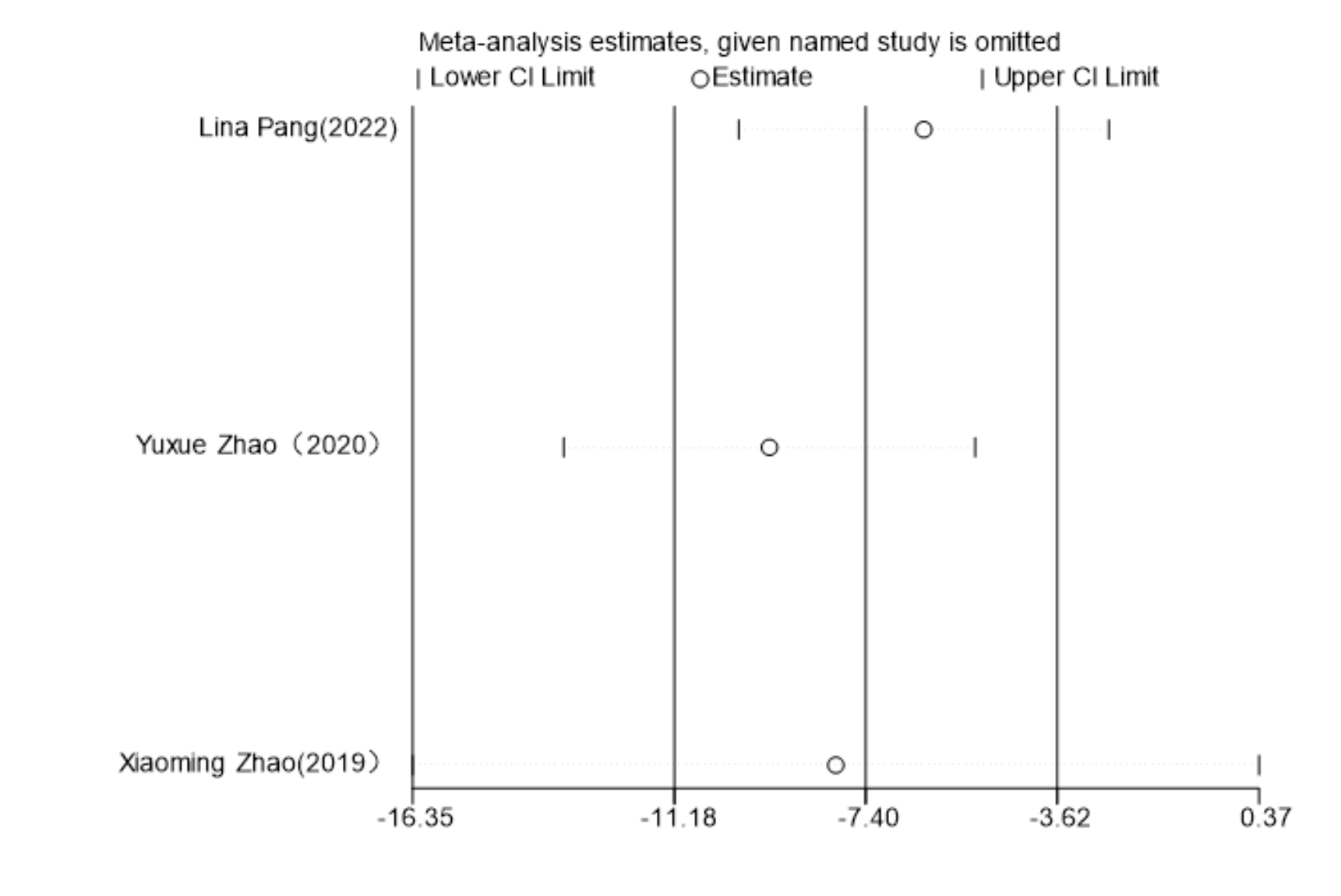

TNF-α - Sensitivity Analyses

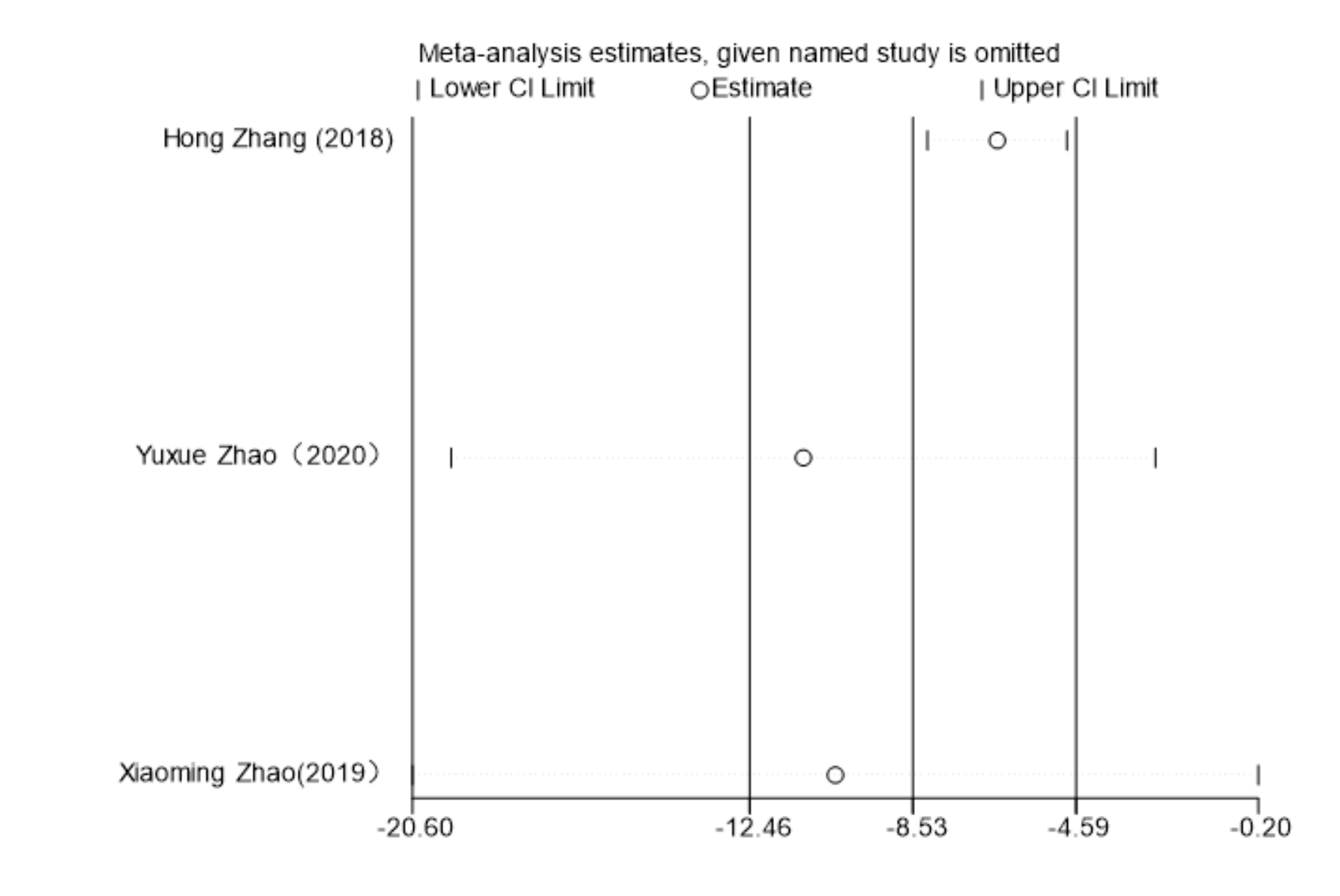

IL-1β - Sensitivity Analyses

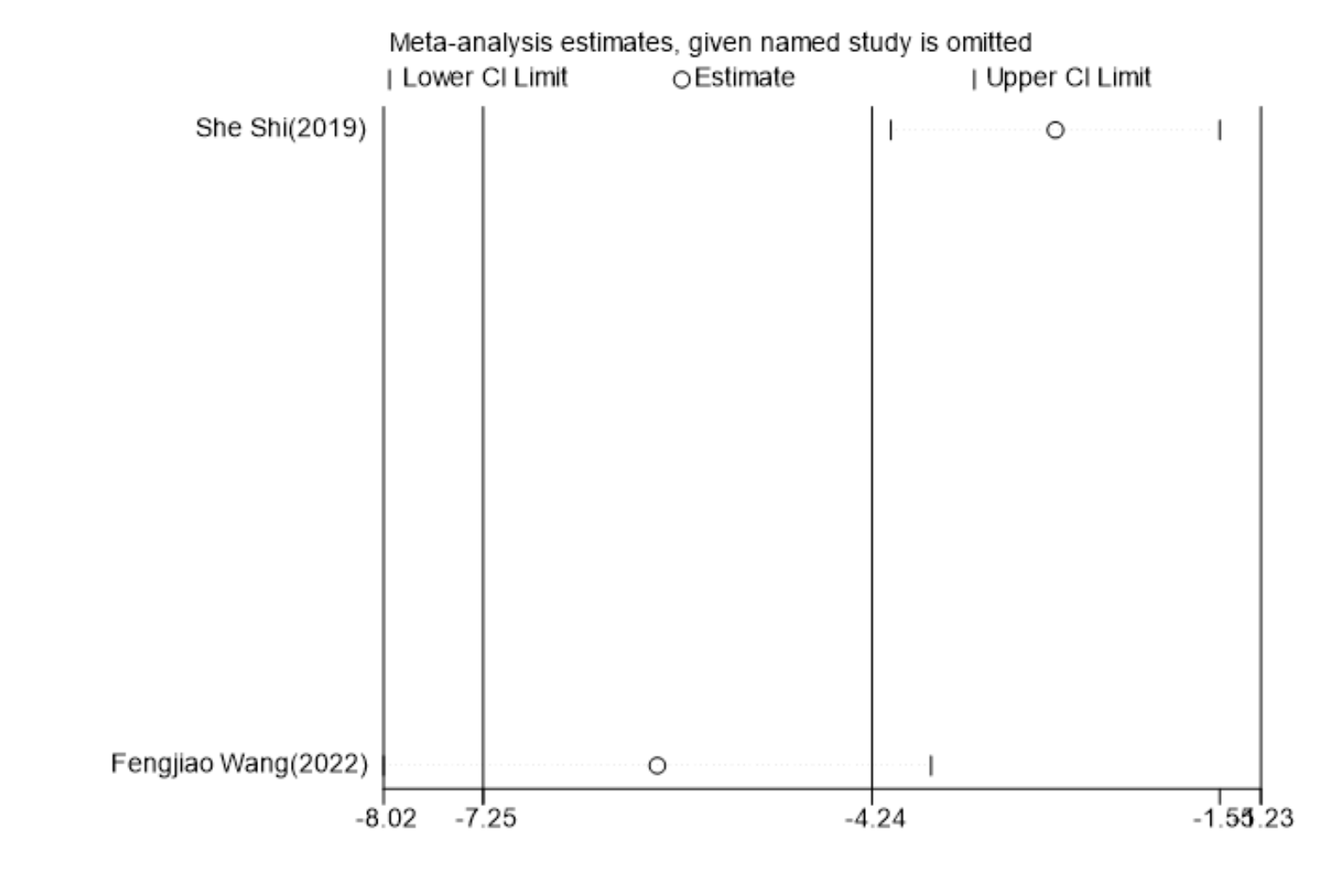

TRPA1- Sensitivity Analyses

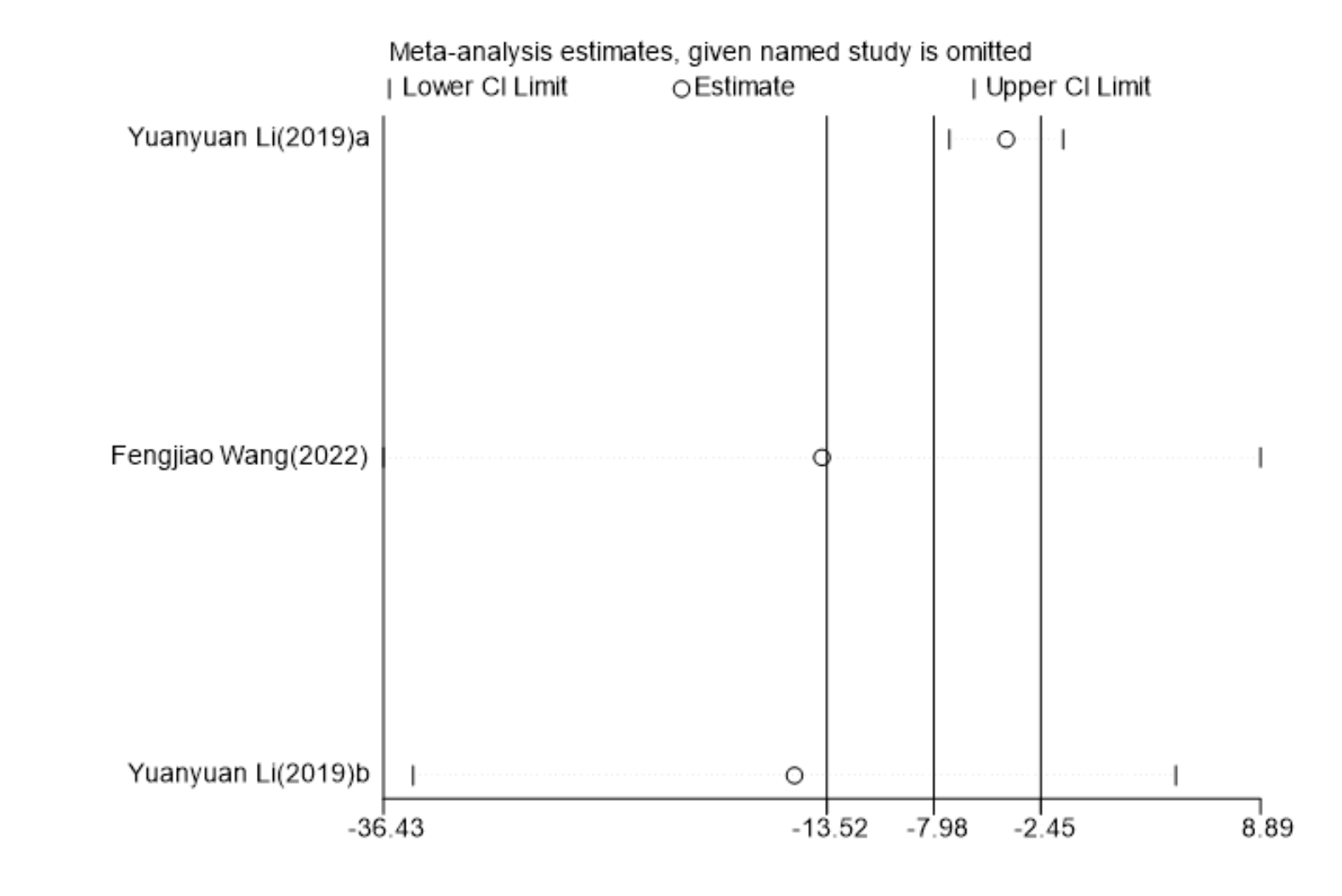

TRPV1- Sensitivity Analyses

D
Subgroup Analyses

[Yuanyuan Li 2019(a): reference 18; Yuanyuan Li 2019(b): reference 23]

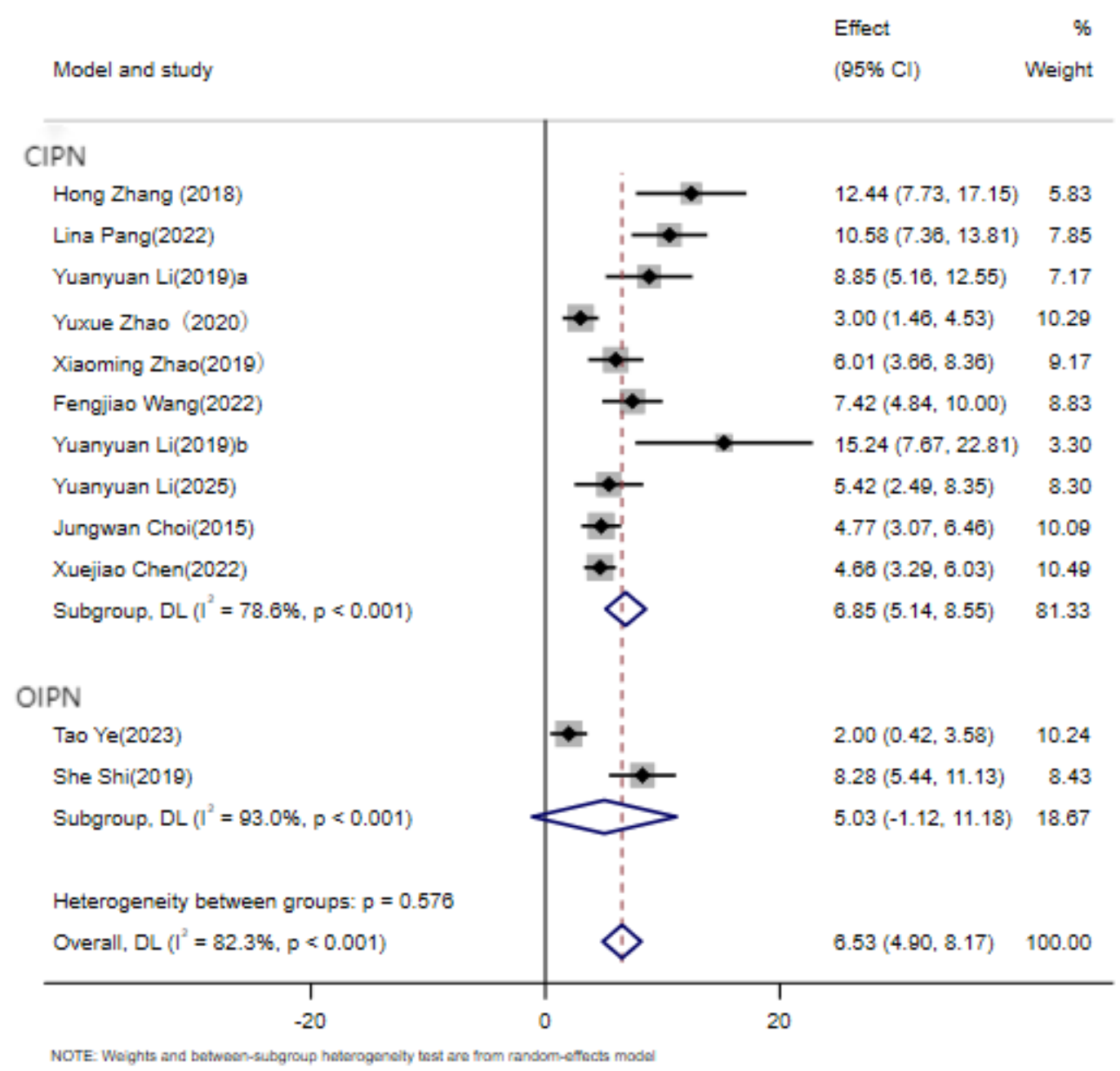

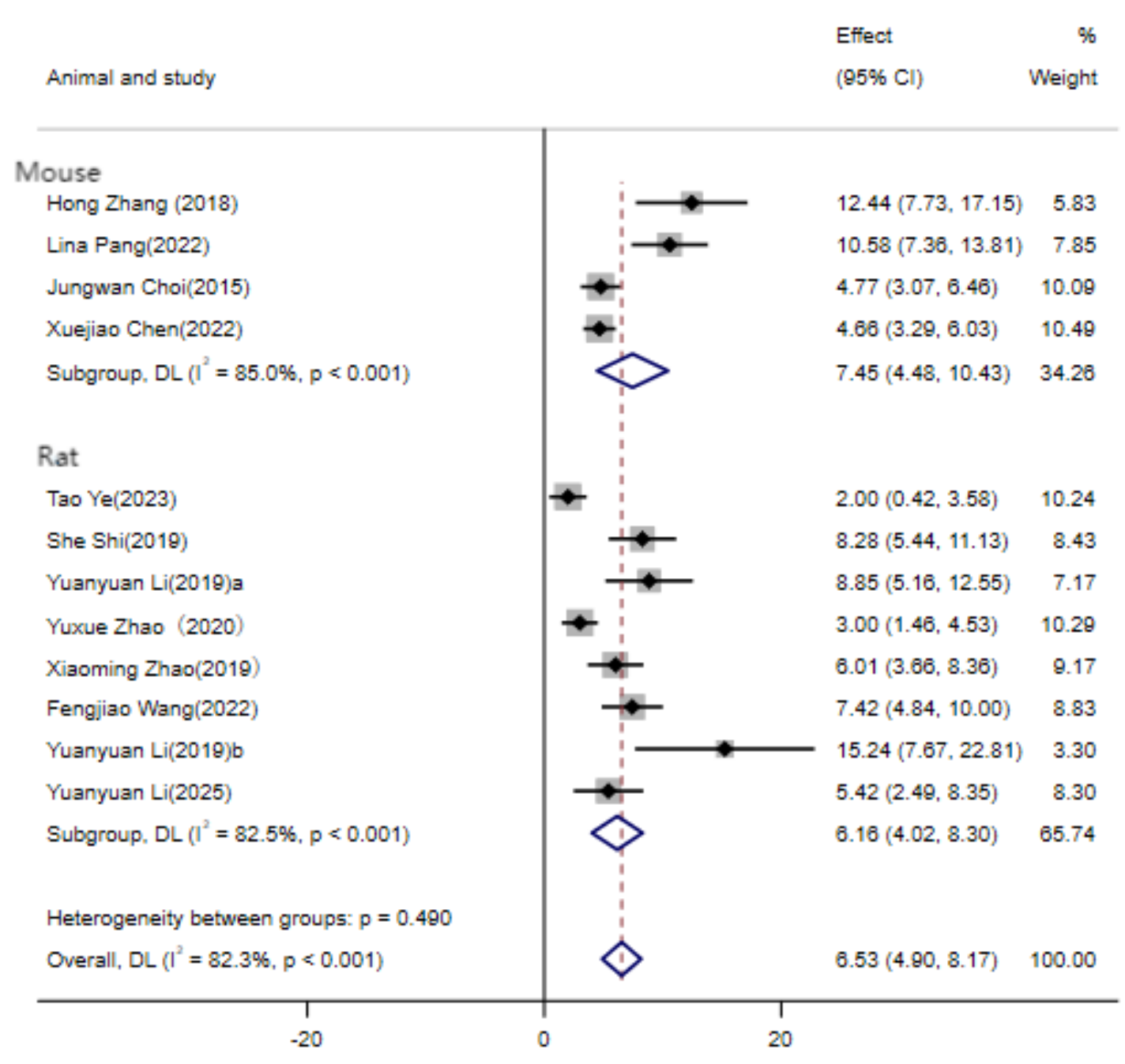

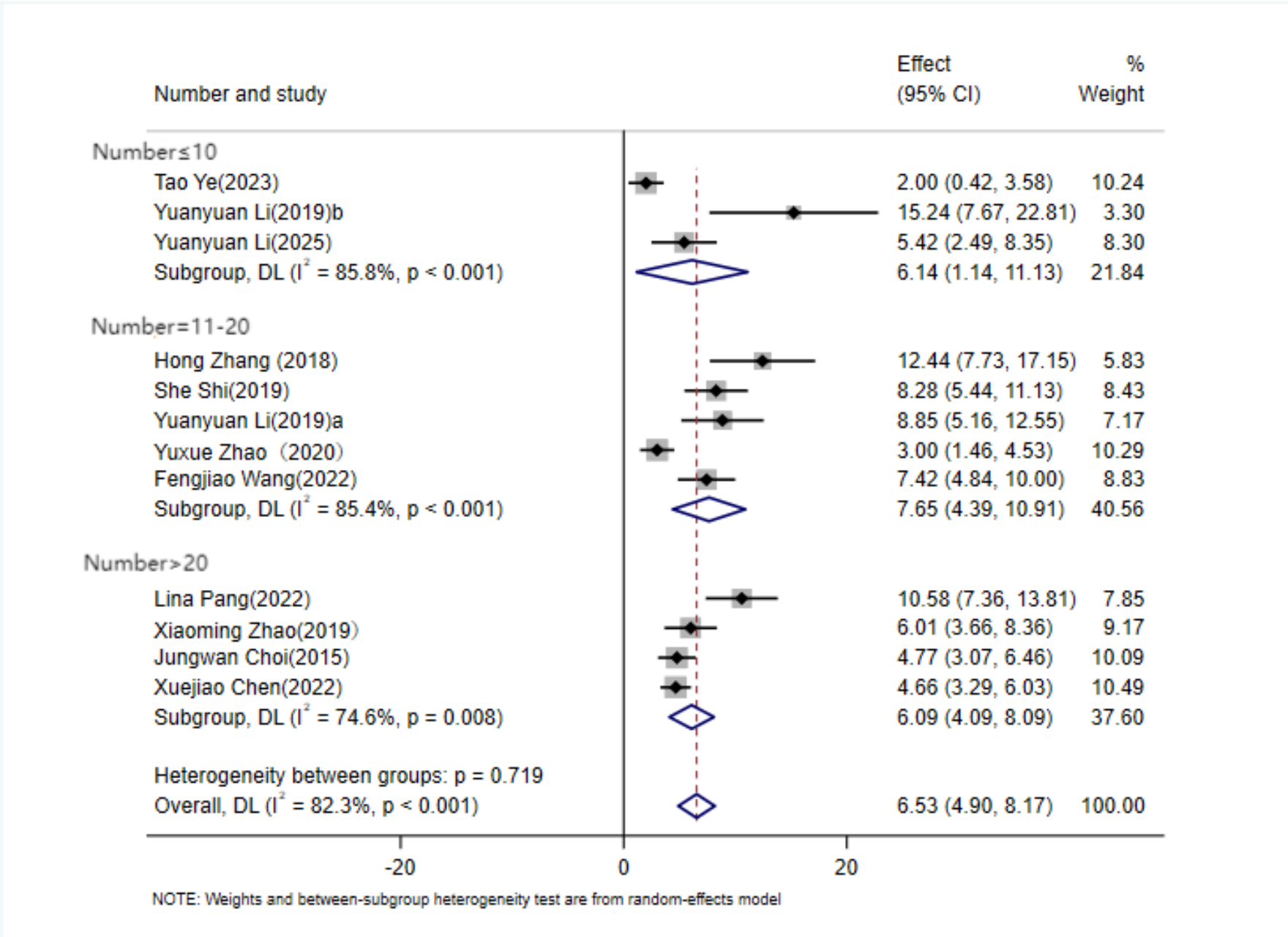
Number and study
Effect (95% CI)
% Weight
Number≤10
Tao Ye(2023) 2.00 (0.42, 3.58) 10.24
Yuanyuan Li(2019)b 15.24 (7.67, 22.81) 3.30
Yuanyuan Li(2025) 5.42 (2.49, 8.35) 8.30
Subgroup, DL (I² = 85.8%, p < 0.001) 6.14 (1.14, 11.13) 21.84
Number=11-20
Hong Zhang (2018) 12.44 (7.73, 17.15) 5.83
She Shi(2019) 8.28 (5.44, 11.13) 8.43
Yuanyuan Li(2019)a 8.85 (5.16, 12.55) 7.17
Yuxue Zhao（2020） 3.00 (1.46, 4.53) 10.29
Fengjiao Wang(2022) 7.42 (4.84, 10.00) 8.83
Subgroup, DL (I² = 85.4%, p < 0.001) 7.65 (4.39, 10.91) 40.56
Number>20
Lina Pang(2022) 10.58 (7.36, 13.81) 7.85
Xiaoming Zhao(2019) 6.01 (3.66, 8.36) 9.17
Jungwan Choi(2015) 4.77 (3.07, 6.46) 10.09
Xuejiao Chen(2022) 4.66 (3.29, 6.03) 10.49
Subgroup, DL (I² = 74.6%, p = 0.008) 6.09 (4.09, 8.09) 37.60
Heterogeneity between groups: p = 0.719
Overall, DL (I² = 82.3%, p < 0.001) 6.53 (4.90, 8.17) 100.00
-20
0
20
NOTE: Weights and between-subgroup heterogeneity test are from random-effects model

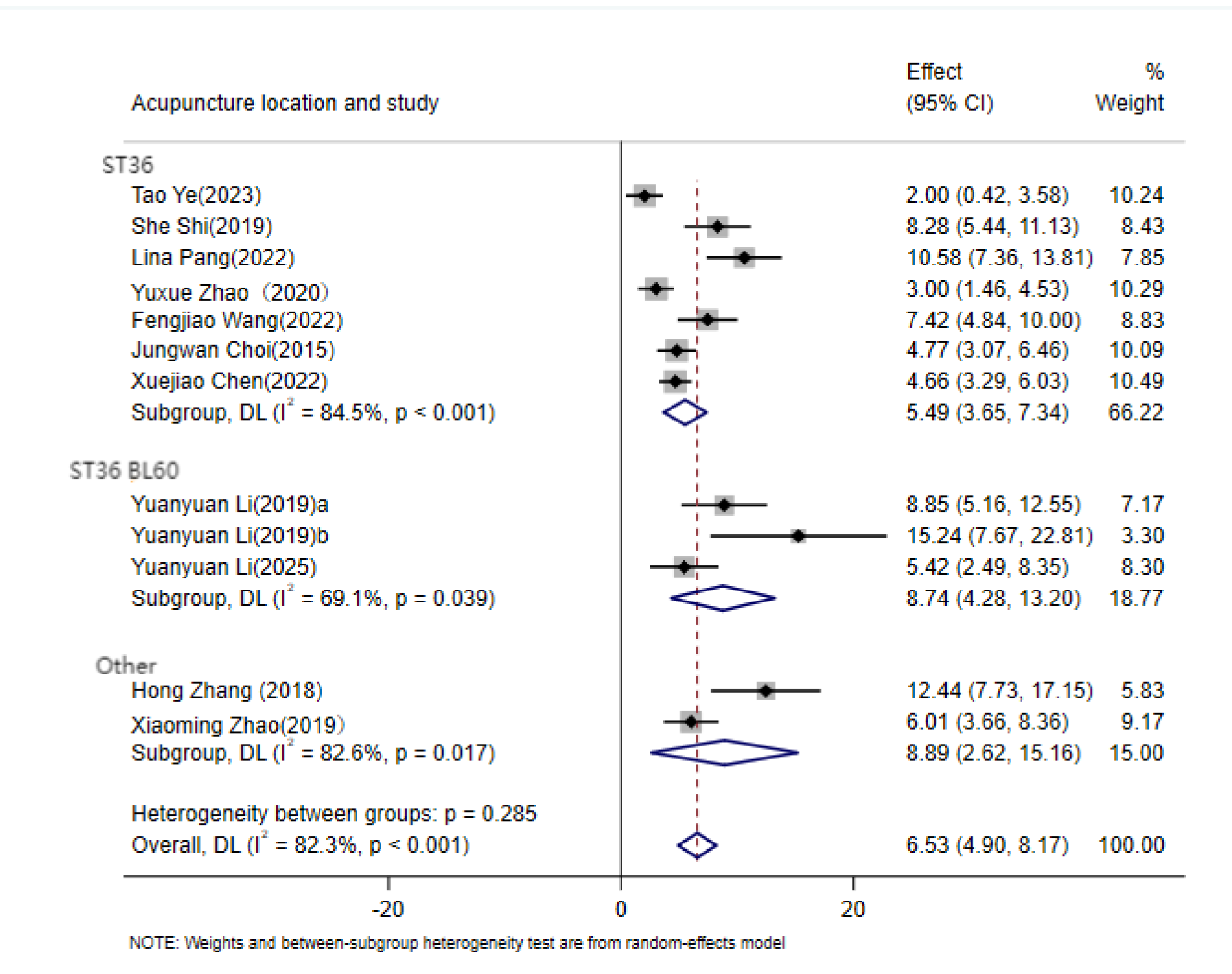
Acupuncture location and study
Effect (95% CI)
% Weight
ST36
Tao Ye(2023) 2.00 (0.42, 3.58) 10.24
She Shi(2019) 8.28 (5.44, 11.13) 8.43
Lina Pang(2022) 10.58 (7.36, 13.81) 7.85
Yuxue Zhao（2020） 3.00 (1.46, 4.53) 10.29
Fengjiao Wang(2022) 7.42 (4.84, 10.00) 8.83
Jungwan Choi(2015) 4.77 (3.07, 6.46) 10.09
Xuejiao Chen(2022) 4.66 (3.29, 6.03) 10.49
Subgroup, DL (I² = 84.5%, p < 0.001) 5.49 (3.65, 7.34) 66.22
ST36 BL60
Yuanyuan Li(2019)a 8.85 (5.16, 12.55) 7.17
Yuanyuan Li(2019)b 15.24 (7.67, 22.81) 3.30
Yuanyuan Li(2025) 5.42 (2.49, 8.35) 8.30
Subgroup, DL (I² = 69.1%, p = 0.039) 8.74 (4.28, 13.20) 18.77
Other
Hong Zhang (2018) 12.44 (7.73, 17.15) 5.83
Xiaoming Zhao(2019) 6.01 (3.66, 8.36) 9.17
Subgroup, DL (I² = 82.6%, p = 0.017) 8.89 (2.62, 15.16) 15.00
Heterogeneity between groups: p = 0.285
Overall, DL (I² = 82.3%, p < 0.001) 6.53 (4.90, 8.17) 100.00
-20
0
20
NOTE: Weights and between-subgroup heterogeneity test are from random-effects model

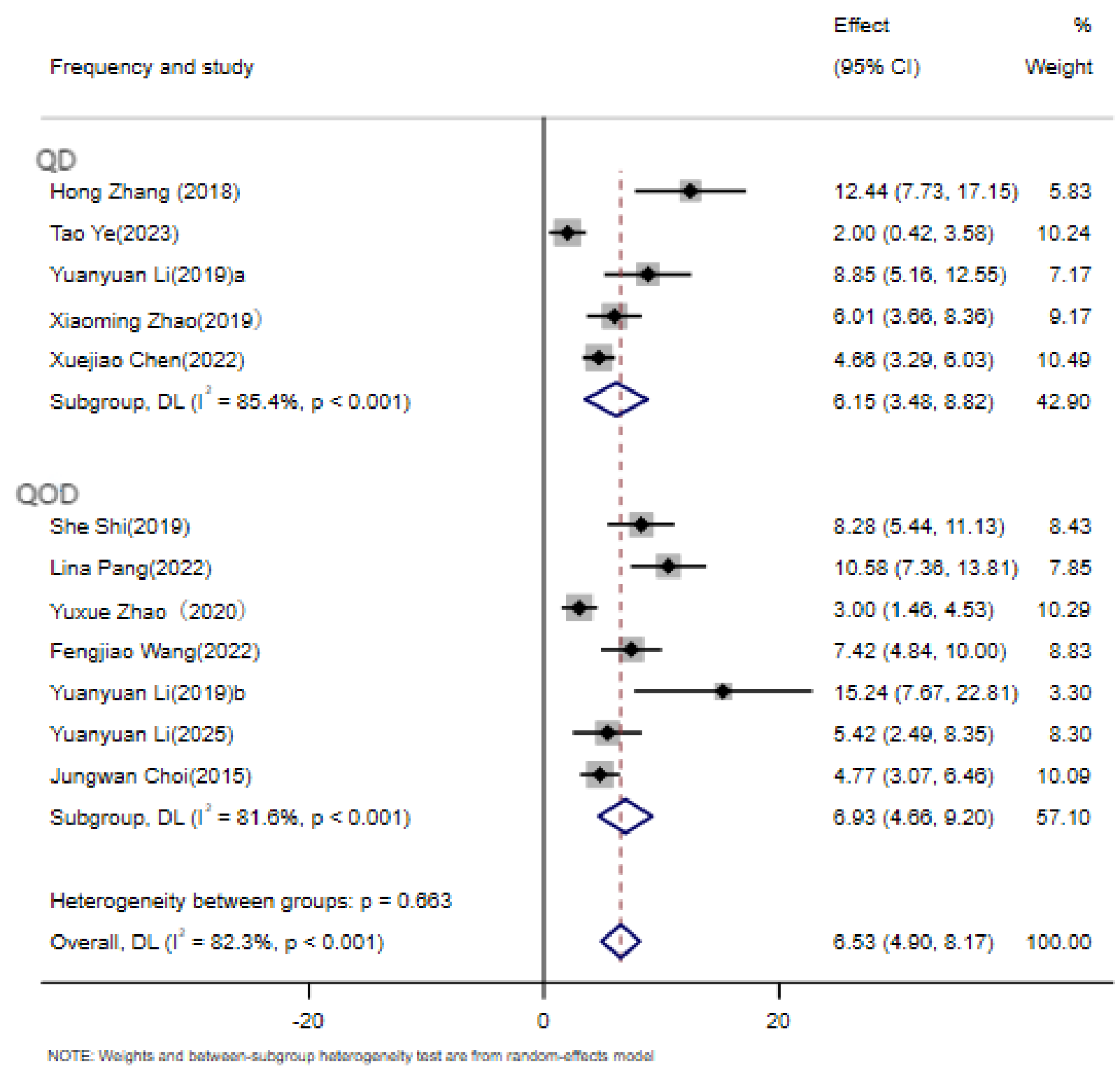
Frequency and study
Effect (95% CI)
% Weight
QD
Hong Zhang (2018) 12.44 (7.73, 17.15) 5.83
Tao Ye(2023) 2.00 (0.42, 3.58) 10.24
Yuanyuan Li(2019)a 8.85 (5.16, 12.55) 7.17
Xiaoming Zhao(2019) 6.01 (3.66, 8.36) 9.17
Xuejiao Chen(2022) 4.66 (3.29, 6.03) 10.49
Subgroup, DL (I² = 85.4%, p < 0.001) 6.15 (3.48, 8.82) 42.90
QOD
She Shi(2019) 8.28 (5.44, 11.13) 8.43
Lina Pang(2022) 10.58 (7.36, 13.81) 7.85
Yuxue Zhao（2020） 3.00 (1.46, 4.53) 10.29
Fengjiao Wang(2022) 7.42 (4.84, 10.00) 8.83
Yuanyuan Li(2019)b 15.24 (7.67, 22.81) 3.30
Yuanyuan Li(2025) 5.42 (2.49, 8.35) 8.30
Jungwan Choi(2015) 4.77 (3.07, 6.46) 10.09
Subgroup, DL (I² = 81.6%, p < 0.001) 6.93 (4.66, 9.20) 57.10
Heterogeneity between groups: p = 0.663
Overall, DL (I² = 82.3%, p < 0.001) 6.53 (4.90, 8.17) 100.00
-20
0
20
NOTE: Weights and between-subgroup heterogeneity test are from random-effects model

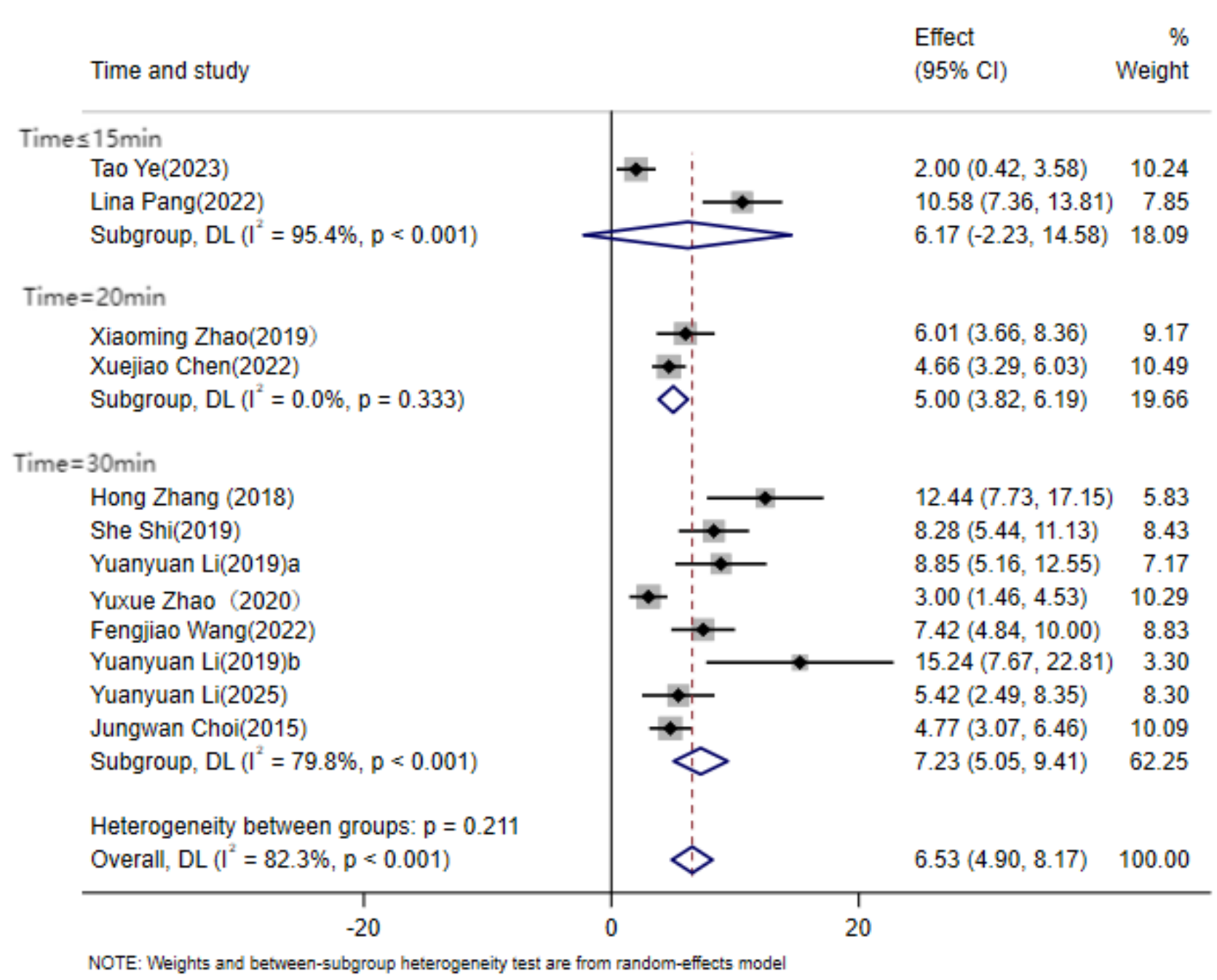
Time and study
Effect (95% CI)
% Weight
Time≤15min
Tao Ye(2023) 2.00 (0.42, 3.58) 10.24
Lina Pang(2022) 10.58 (7.36, 13.81) 7.85
Subgroup, DL (I² = 95.4%, p < 0.001) 6.17 (-2.23, 14.58) 18.09
Time=20min
Xiaoming Zhao(2019) 6.01 (3.66, 8.36) 9.17
Xuejiao Chen(2022) 4.66 (3.29, 6.03) 10.49
Subgroup, DL (I² = 0.0%, p = 0.333) 5.00 (3.82, 6.19) 19.66
Time=30min
Hong Zhang (2018) 12.44 (7.73, 17.15) 5.83
She Shi(2019) 8.28 (5.44, 11.13) 8.43
Yuanyuan Li(2019)a 8.85 (5.16, 12.55) 7.17
Yuxue Zhao（2020） 3.00 (1.46, 4.53) 10.29
Fengjiao Wang(2022) 7.42 (4.84, 10.00) 8.83
Yuanyuan Li(2019)b 15.24 (7.67, 22.81) 3.30
Yuanyuan Li(2025) 5.42 (2.49, 8.35) 8.30
Jungwan Choi(2015) 4.77 (3.07, 6.46) 10.09
Subgroup, DL (I² = 79.8%, p < 0.001) 7.23 (5.05, 9.41) 62.25
Heterogeneity between groups: p = 0.211
Overall, DL (I² = 82.3%, p < 0.001) 6.53 (4.90, 8.17) 100.00
-20
0
20
NOTE: Weights and between-subgroup heterogeneity test are from random-effects model

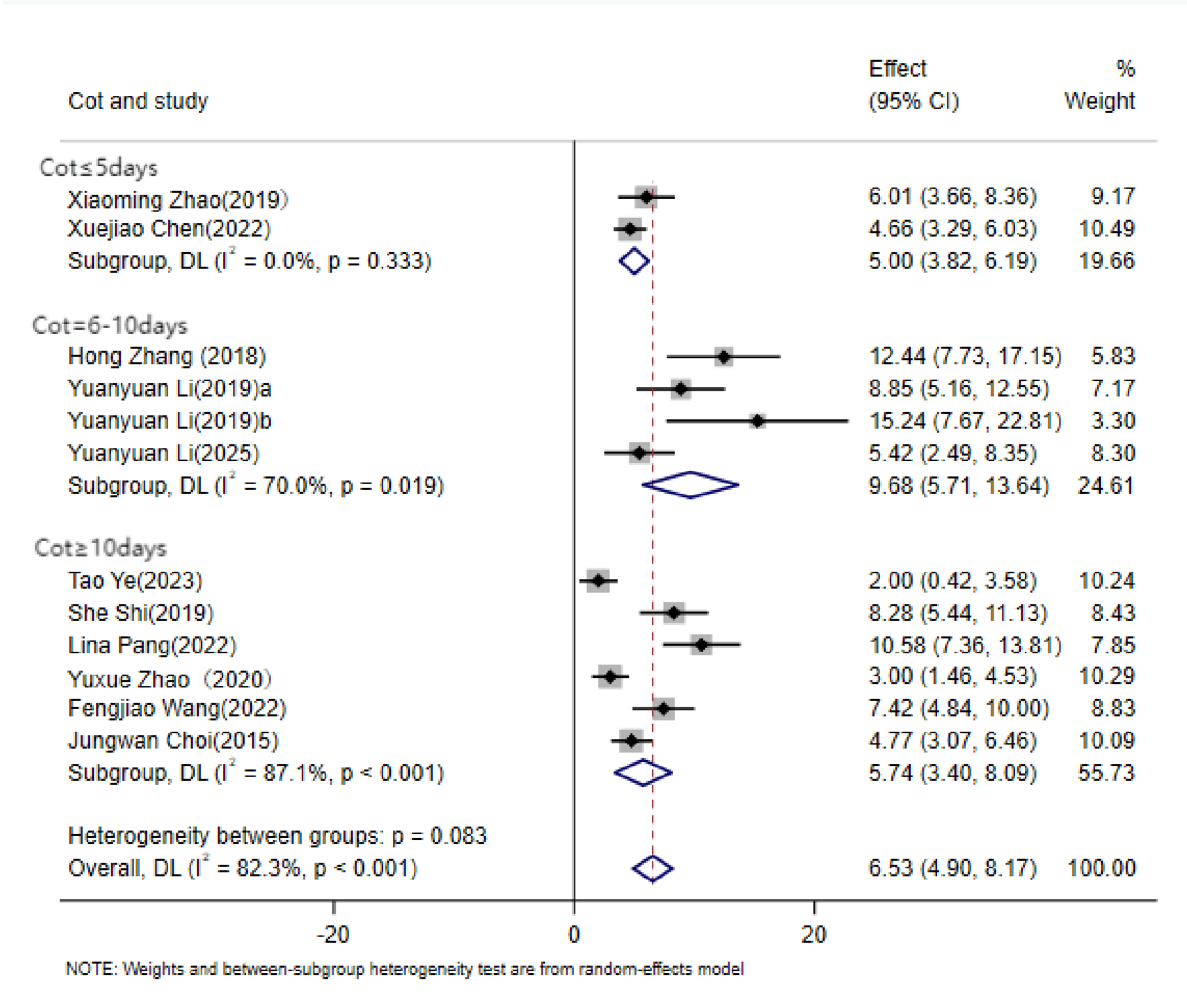
Cot and study
Effect (95% CI)
% Weight
Cot≤5days
Xiaoming Zhao(2019) 6.01 (3.66, 8.36) 9.17
Xuejiao Chen(2022) 4.66 (3.29, 6.03) 10.49
Subgroup, DL (I² = 0.0%, p = 0.333) 5.00 (3.82, 6.19) 19.66
Cot=6-10days
Hong Zhang (2018) 12.44 (7.73, 17.15) 5.83
Yuanyuan Li(2019)a 8.85 (5.16, 12.55) 7.17
Yuanyuan Li(2019)b 15.24 (7.67, 22.81) 3.30
Yuanyuan Li(2025) 5.42 (2.49, 8.35) 8.30
Subgroup, DL (I² = 70.0%, p = 0.019) 9.68 (5.71, 13.64) 24.61
Cot≥10days
Tao Ye(2023) 2.00 (0.42, 3.58) 10.24
She Shi(2019) 8.28 (5.44, 11.13) 8.43
Lina Pang(2022) 10.58 (7.36, 13.81) 7.85
Yuxue Zhao（2020） 3.00 (1.46, 4.53) 10.29
Fengjiao Wang(2022) 7.42 (4.84, 10.00) 8.83
Jungwan Choi(2015) 4.77 (3.07, 6.46) 10.09
Subgroup, DL (I² = 87.1%, p < 0.001) 5.74 (3.40, 8.09) 55.73
Heterogeneity between groups: p = 0.083
Overall, DL (I² = 82.3%, p < 0.001) 6.53 (4.90, 8.17) 100.00
-20
0
20
NOTE: Weights and between-subgroup heterogeneity test are from random-effects model

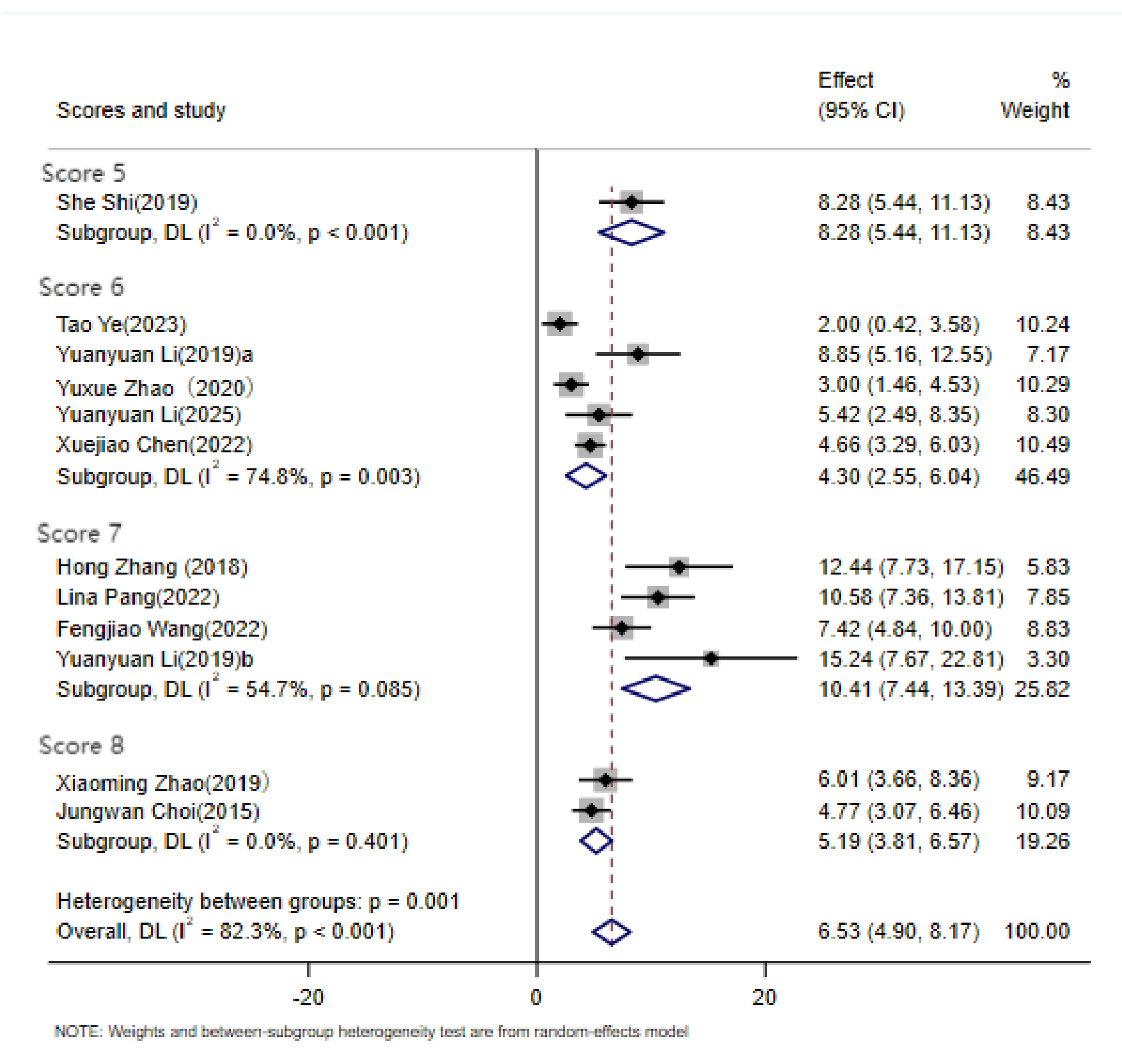
Scores and study
Effect (95% CI)
% Weight
Score 5
She Shi(2019) 8.28 (5.44, 11.13) 8.43
Subgroup, DL (I² = 0.0%, p < 0.001) 8.28 (5.44, 11.13) 8.43
Score 6
Tao Ye(2023) 2.00 (0.42, 3.58) 10.24
Yuanyuan Li(2019)a 8.85 (5.16, 12.55) 7.17
Yuxue Zhao（2020） 3.00 (1.46, 4.53) 10.29
Yuanyuan Li(2025) 5.42 (2.49, 8.35) 8.30
Xuejiao Chen(2022) 4.66 (3.29, 6.03) 10.49
Subgroup, DL (I² = 74.8%, p = 0.003) 4.30 (2.55, 6.04) 46.49
Score 7
Hong Zhang (2018) 12.44 (7.73, 17.15) 5.83
Lina Pang(2022) 10.58 (7.36, 13.81) 7.85
Fengjiao Wang(2022) 7.42 (4.84, 10.00) 8.83
Yuanyuan Li(2019)b 15.24 (7.67, 22.81) 3.30
Subgroup, DL (I² = 54.7%, p = 0.085) 10.41 (7.44, 13.39) 25.82
Score 8
Xiaoming Zhao(2019) 6.01 (3.66, 8.36) 9.17
Jungwan Choi(2015) 4.77 (3.07, 6.46) 10.09
Subgroup, DL (I² = 0.0%, p = 0.401) 5.19 (3.81, 6.57) 19.26
Heterogeneity between groups: p = 0.001
Overall, DL (I² = 82.3%, p < 0.001) 6.53 (4.90, 8.17) 100.00
-20
0
20
NOTE: Weights and between-subgroup heterogeneity test are from random-effects model

E
Publication Bias

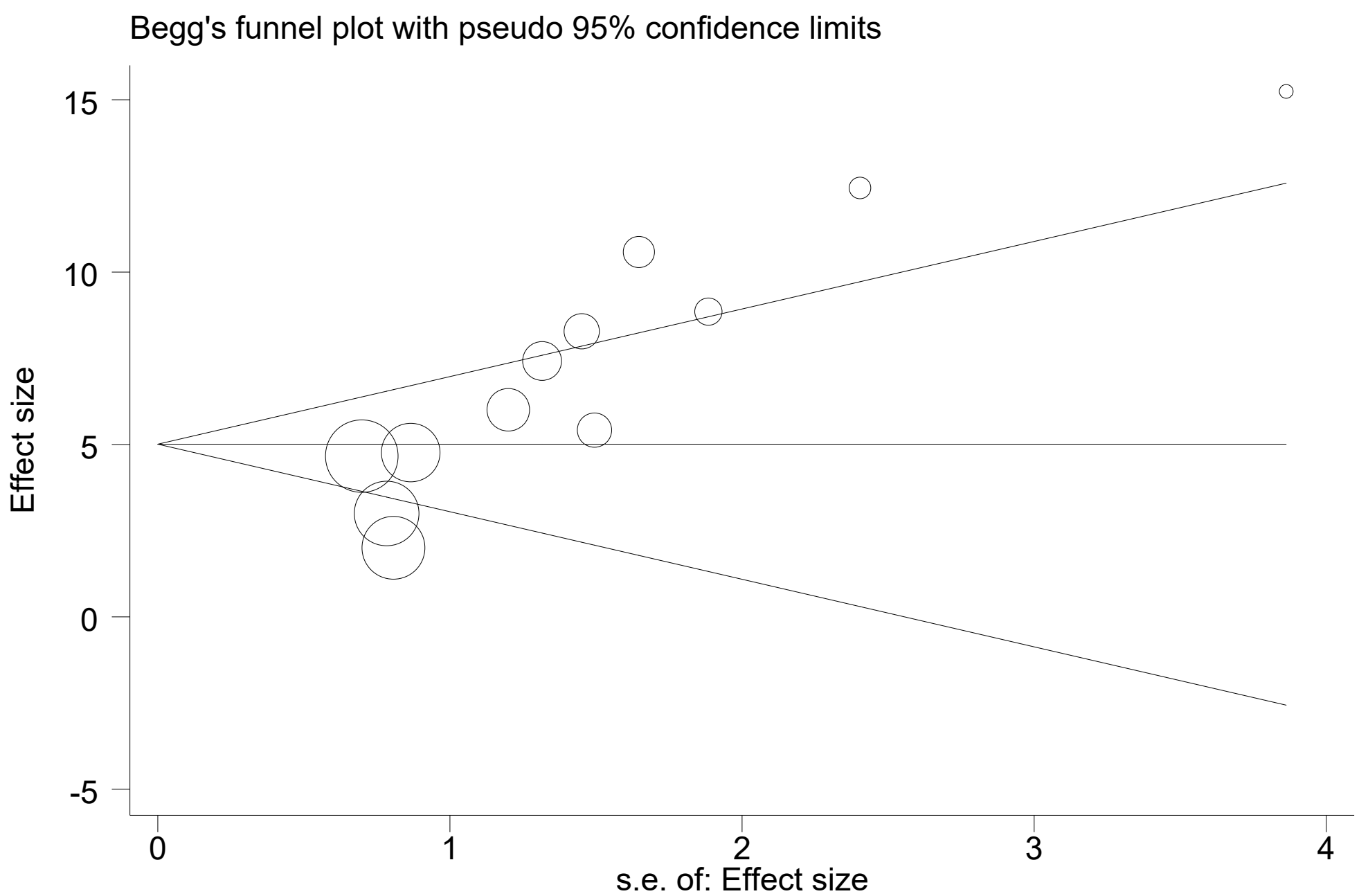

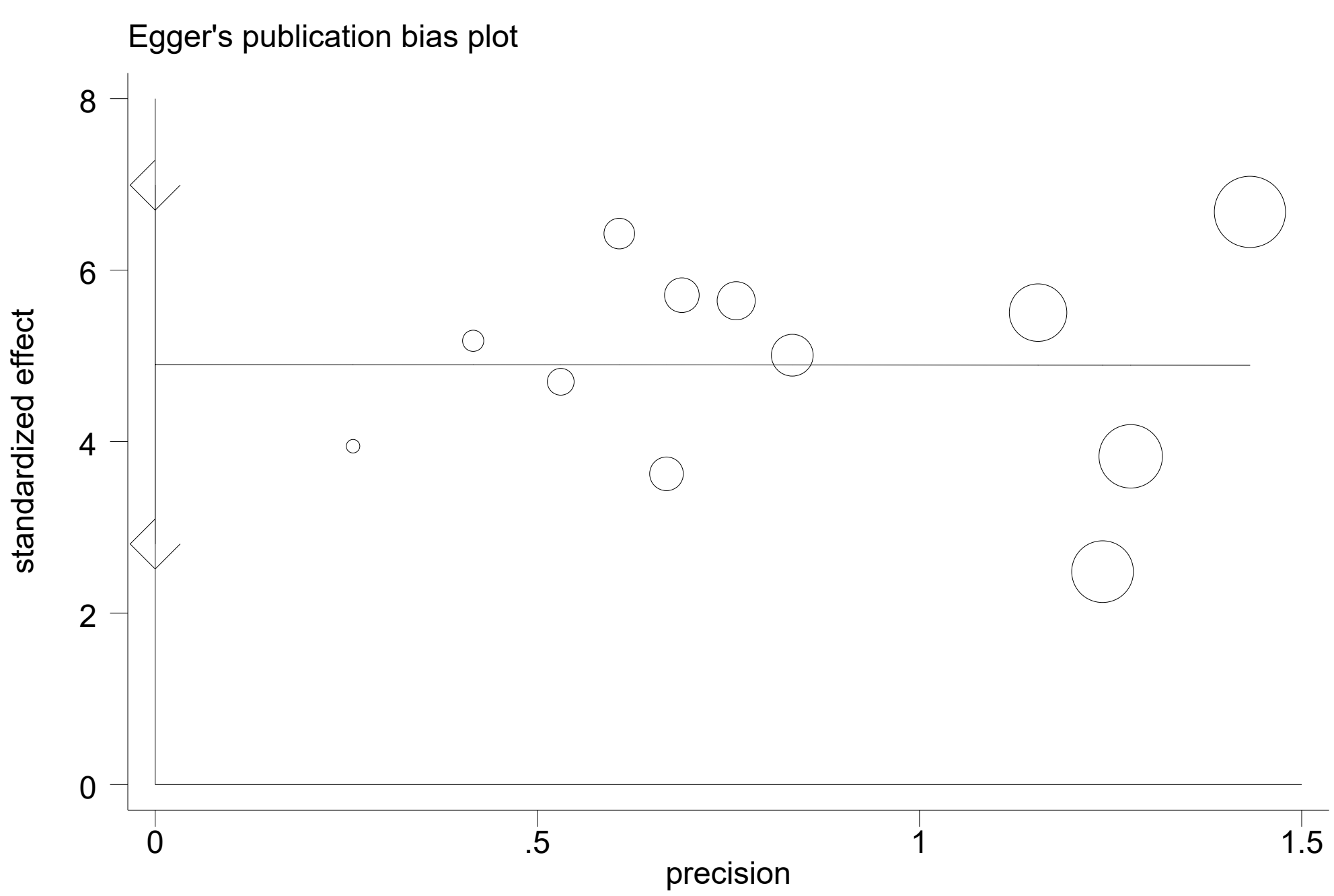

F Data extraction table

[Yuanyuan Li 2019(a): reference 18; Yuanyuan Li 2019(b): reference 23]

Study	EA Group			Model Group		
PWT	n1	mean1	sd1	n2	mean2	sd2
Hong Zhang (2018)	8	8.72	0.56	8	1.50	0.60
Tao Ye (2023)	5	10.80	4.09	5	4.80	1.10
She Shi (2019)	10	19.34	2.64	10	2.52	1.13
Lina Pang (2022)	12	4.62	0.50	12	0.59	0.20
Yuanyuan Li(2019)	7	11.65	1.24	7	3.53	0.38
Yuxue Zhao (2020)	7	10.90	0.80	8	7.74	1.23
Xiaoming Zhao(2019)	9	11.77	1.47	8	3.63	1.21
Fengjiao Wang (2022)	10	19.30	2.64	10	2.38	1.85
Yuanyuan Li (2019)b	5	14.01	0.66	5	3.95	0.66
Yuanyuan Li (2025)	5	8.88	2.17	5	0.42	0.41
Jungwan Choi (2015)	11	10.90	0.59	11	8.42	0.44
Xuejiao Chen (2022)	16	8.28	1.98	16	1.31	0.74
PWL						
Lina Pang (2022)	12	8.70	0.20	12	5.53	0.30
Xiaoming Zhao (2019)	9	11.47	1.28	8	5.30	1.14
Yuanyuan Li (2019)b	5	14.95	1.21	5	9.67	0.55
Jungwan Choi (2015)	11	10.81	0.26	11	8.85	0.29
Cold allodynia						
She Shi (2019)	10	1.81	0.44	10	11.32	1.56
Fengjiao Wang (2022)	10	0.99	0.49	10	8.24	0.40
Nerve conduction velocity						
Tao Ye(2023)	5	36.66	3.13	5	18.34	3.31
Guangwei Cui (2017)	10	27.01	0.83	10	21.17	1.24
Weight gain						
Tao Ye (2023)	5	0.33	0.10	5	0.28	0.08
Yuxue Zhao (2020)	7	0.31	0.04	8	0.35	0.05
Yuanyuan Li(2019)b	5	0.50	0.02	5	0.48	0.03
TNF-α						
Lina Pang (2022)	4	11.57	0.59	4	21.56	0.90
Yuxue Zhao (2020)	7	11.92	1.22	8	18.21	1.56
Xiaoming Zhao (2019)	15	7.57	0.21	12	9.56	0.28
IL-1β						
Hong Zhang (2018)	8	0.70	0.05	8	1.50	0.05
Yuxue Zhao (2020)	7	45.72	1.28	8	55.88	2.19
Xiaoming Zhao(2019)	15	147.94	5.61	12	186.52	4.87
GFAP						
Tao Ye (2023)	5	0.60	0.04	5	0.98	0.08
Yuxue Zhao (2020)	3	0.75	0.10	3	1.24	0.09
Xuejiao Chen (2022)	3	1.24	0.38	3	1.80	0.30
TLR4						
Yuxue Zhao (2020)	4	0.84	0.10	4	1.23	0.11
Yuanyuan Li(2019)b	5	1.02	0.06	5	1.33	0.06
TRPA1						
She Shi (2019)	10	1.32	0.15	10	2.05	0.09
Fengjiao Wang (2022)	10	0.80	0.06	10	1.05	0.11
TRPV1						
Yuanyuan Li (2019)a	7	0.72	0.03	7	1.30	0.01
Fengjiao Wang (2022)	10	0.98	0.04	10	1.31	0.07
Yuanyuan Li (2019)b	5	0.84	0.09	5	1.12	0.11
Nrf2						
Xiaoming Zhao (2019)	10	0.85	0.03	10	0.63	0.04
Fengjiao Wang (2022)	10	0.75	0.25	10	0.41	0.19

G
Stata code

Forest plot

```
metan n1 mean1 sd1 n2 mean2 sd2, label (namevar=study) random cohen
```

Subgroup Analyses

```
metan n1 mean1 sd1 n2 mean2 sd2, label (namevar=study) by (factor) random cohen
```

Sensitivity analyses

```
metaninf n1 mean1 sd1 n2 mean2 sd2, label (namevar=study) random cohen
```

Meta-regression

```
metareg _ES factor, wsse (_seES) bsest(reml)
```

Publication bias

```
metabias6 _ES _seES, graph (begg) gweight
metabias6 _ES _seES, graph (egger) gweight
```

Funnel plot

```
metafunnel _ES _seES
```

Contour-enhanced funnel plot

```
confunnel _ES _seES
```

Trim-and-fill analysis

```
metatrim _ES _seES, reffect funnel
```

H
Forest plots

[Yuanyuan Li 2019(a): reference 18; Yuanyuan Li 2019(b): reference 23]

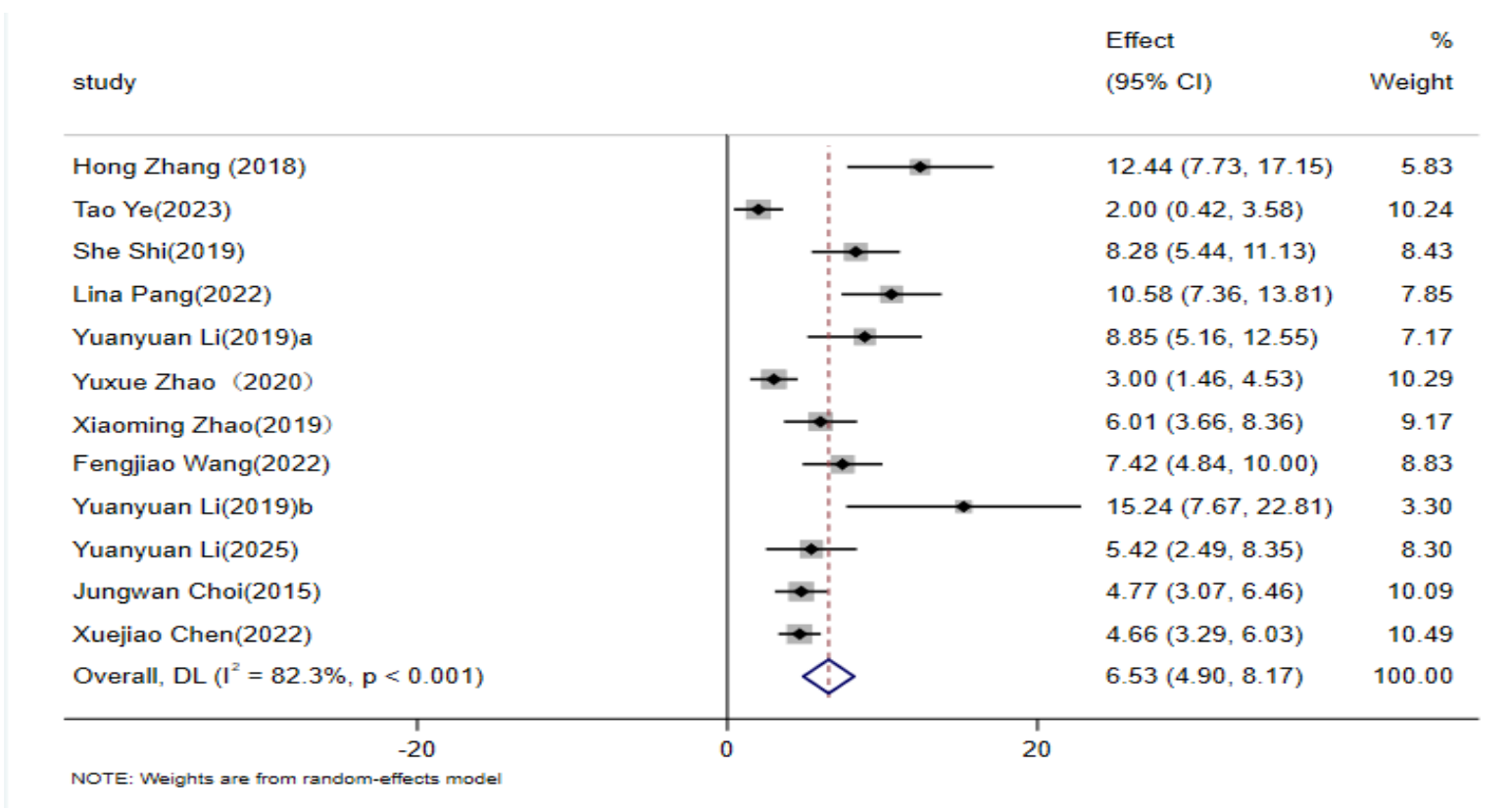

A. PWT Forest plot

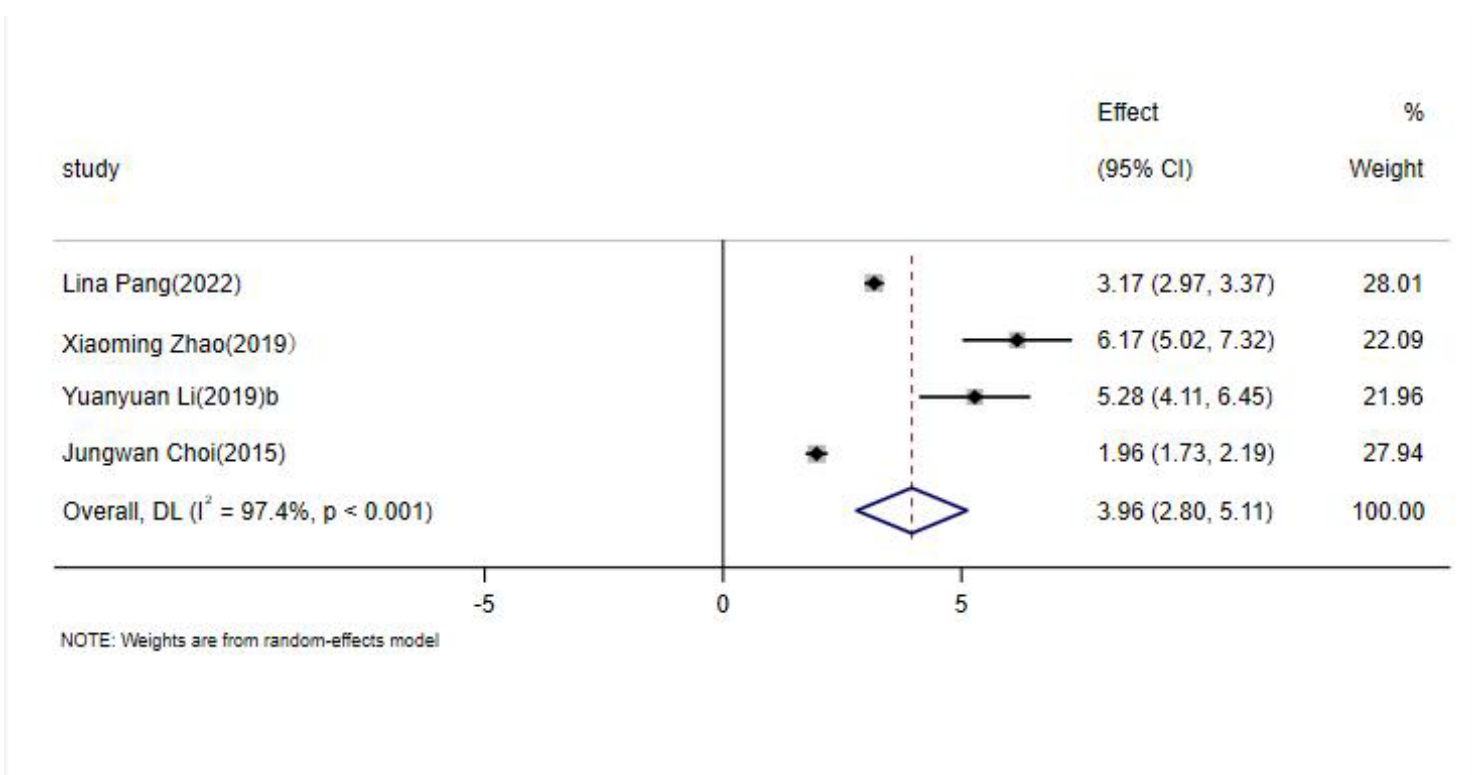

B. PWL Forest plot

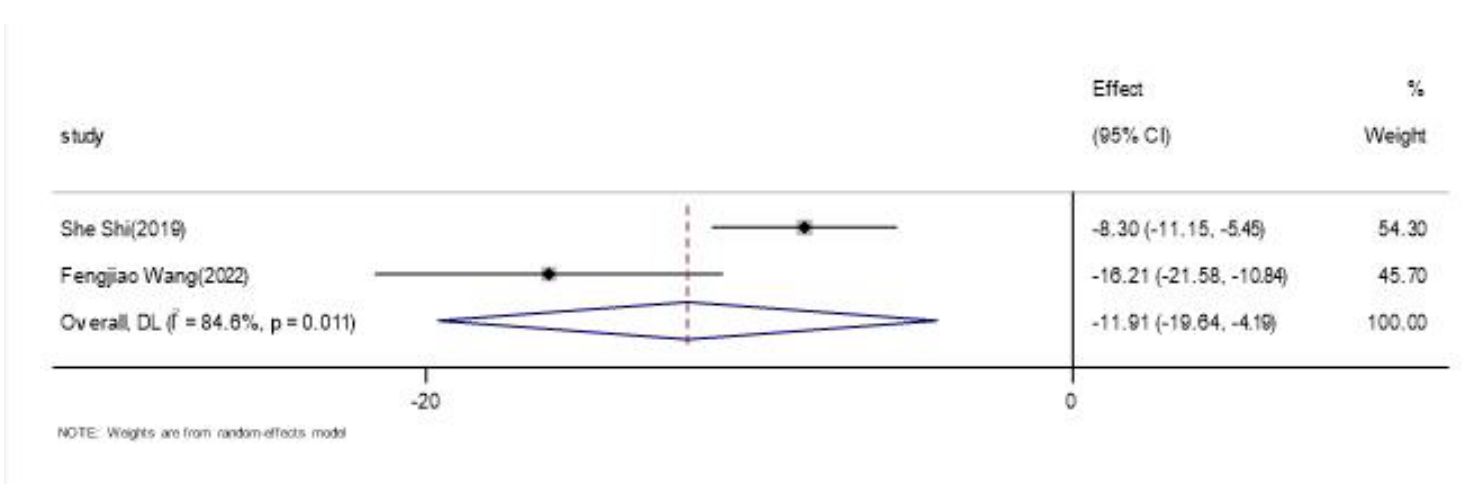

C. Cold allodynia Forest plot

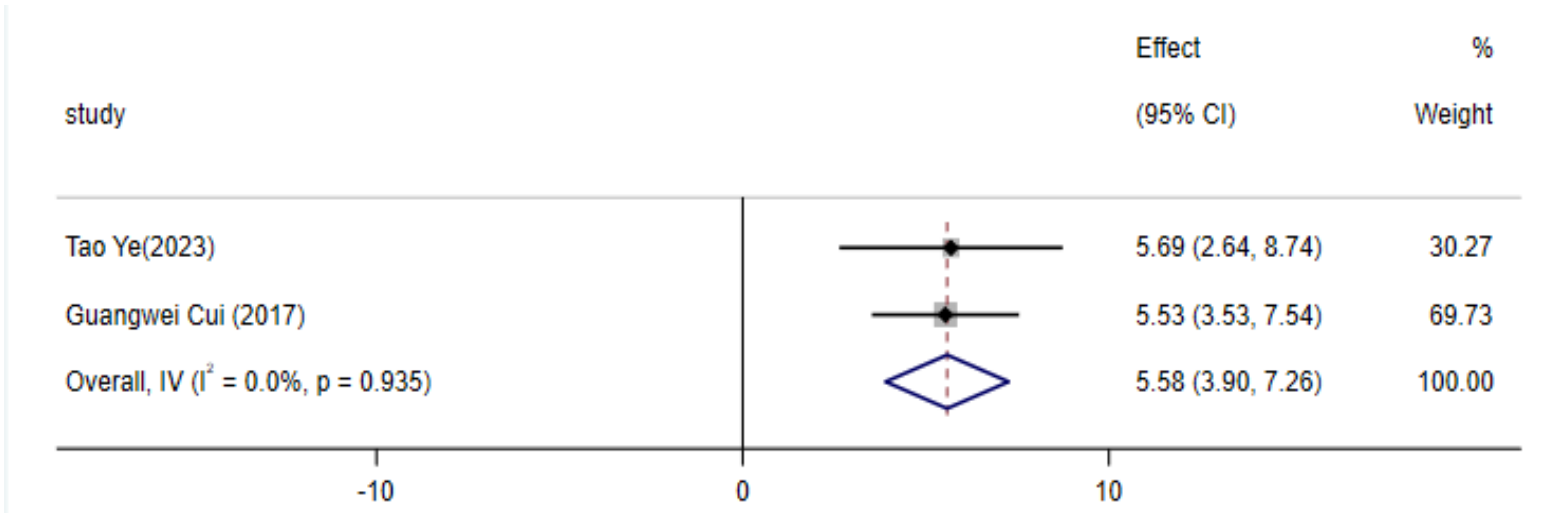

D. NCV Forest plot

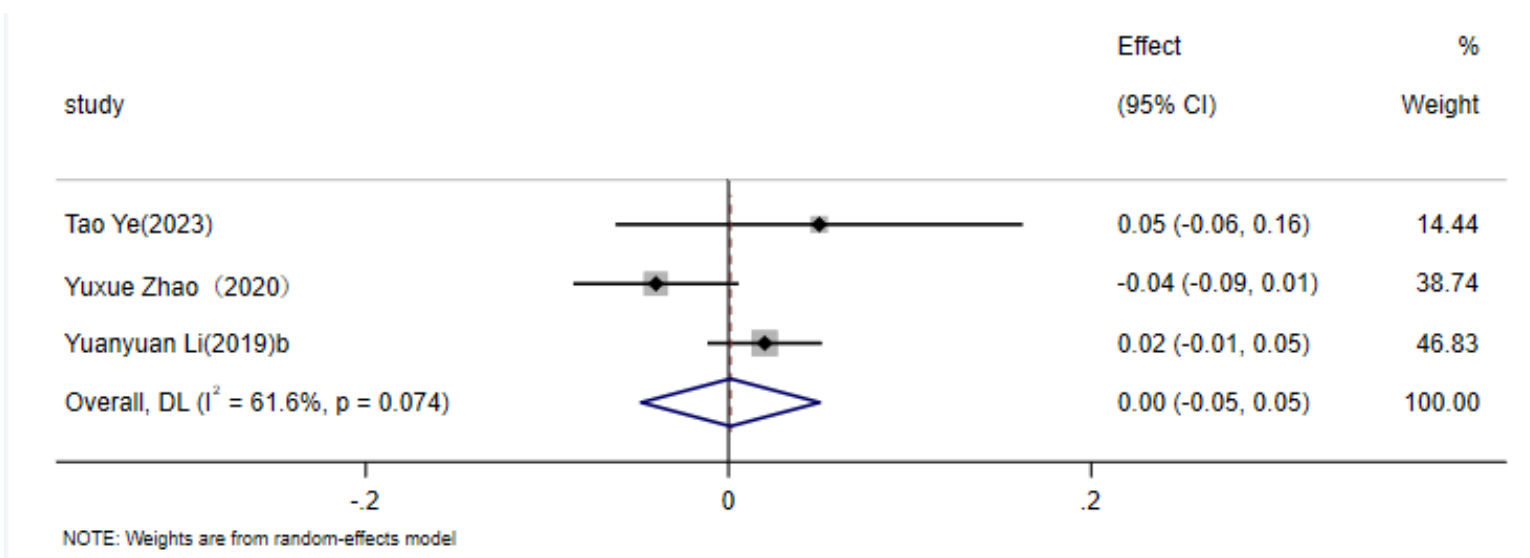

E. Weight gain Forest plot

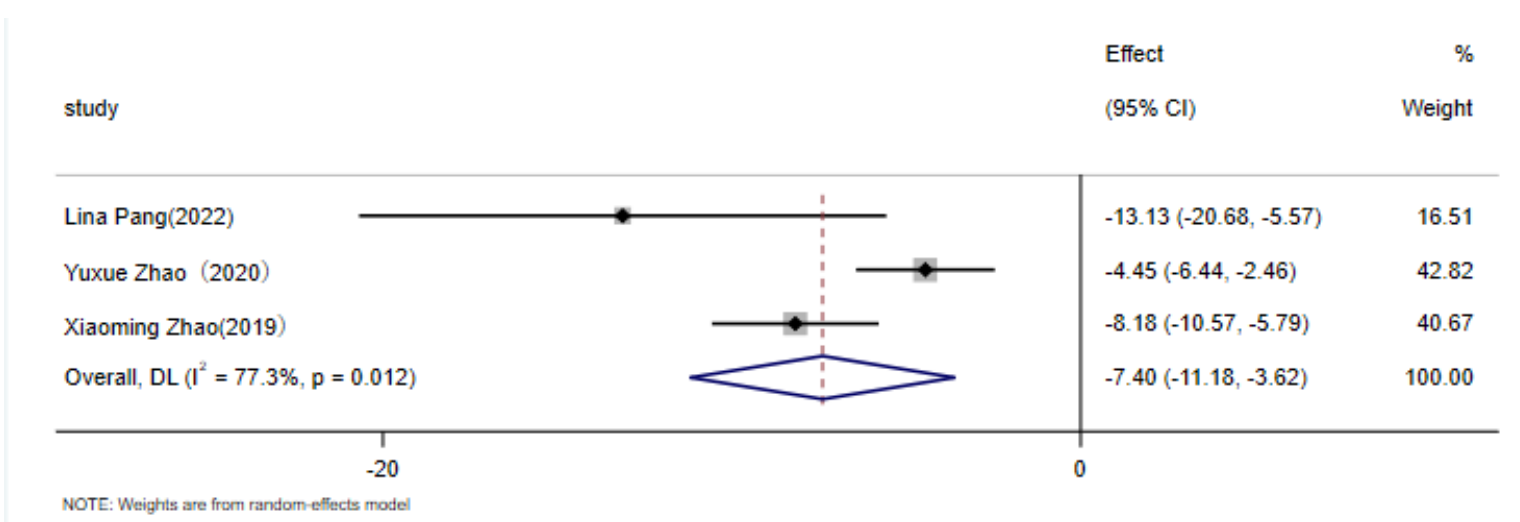

F. TNF-α Forest plot

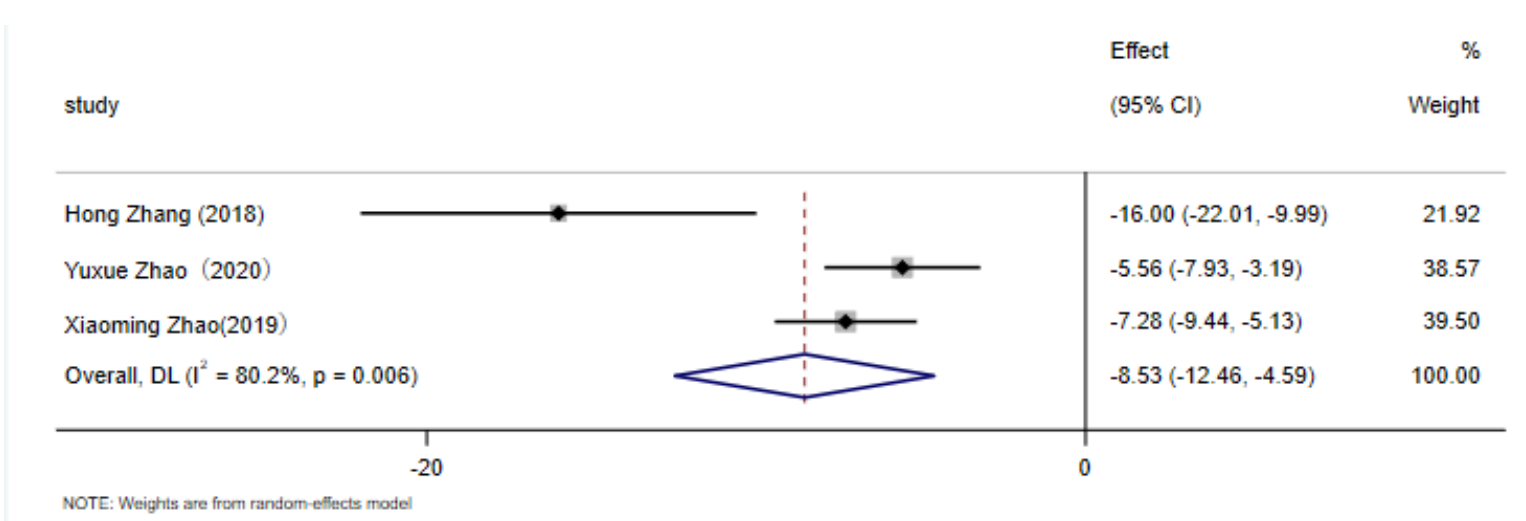

G. IL-1β Forest plot

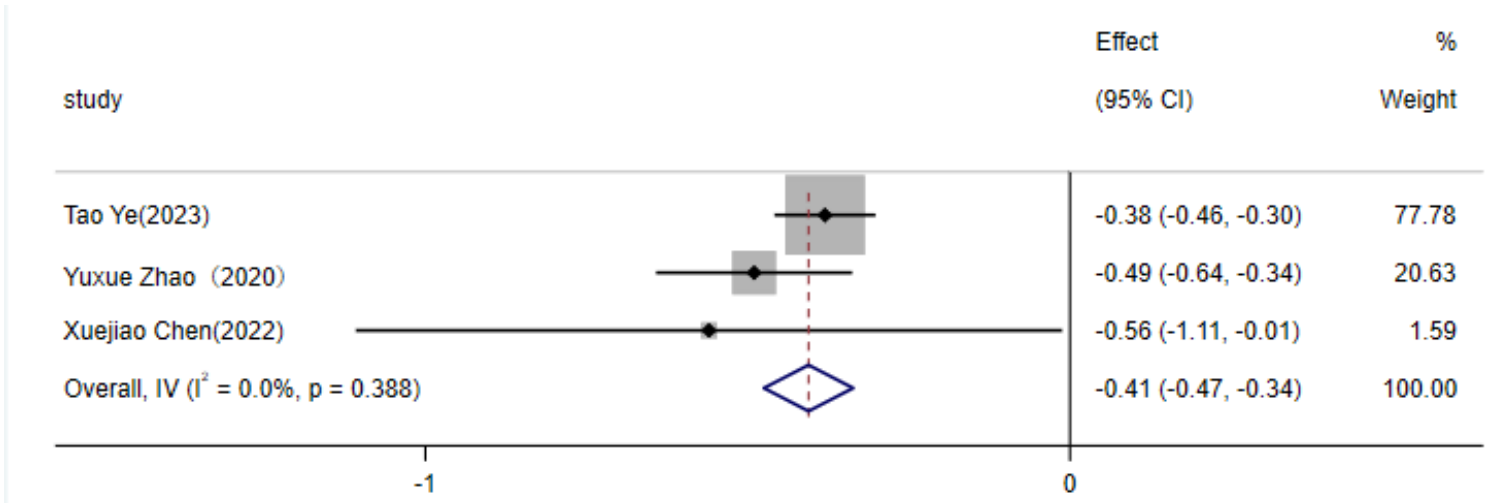

H. GFAP Forest plot

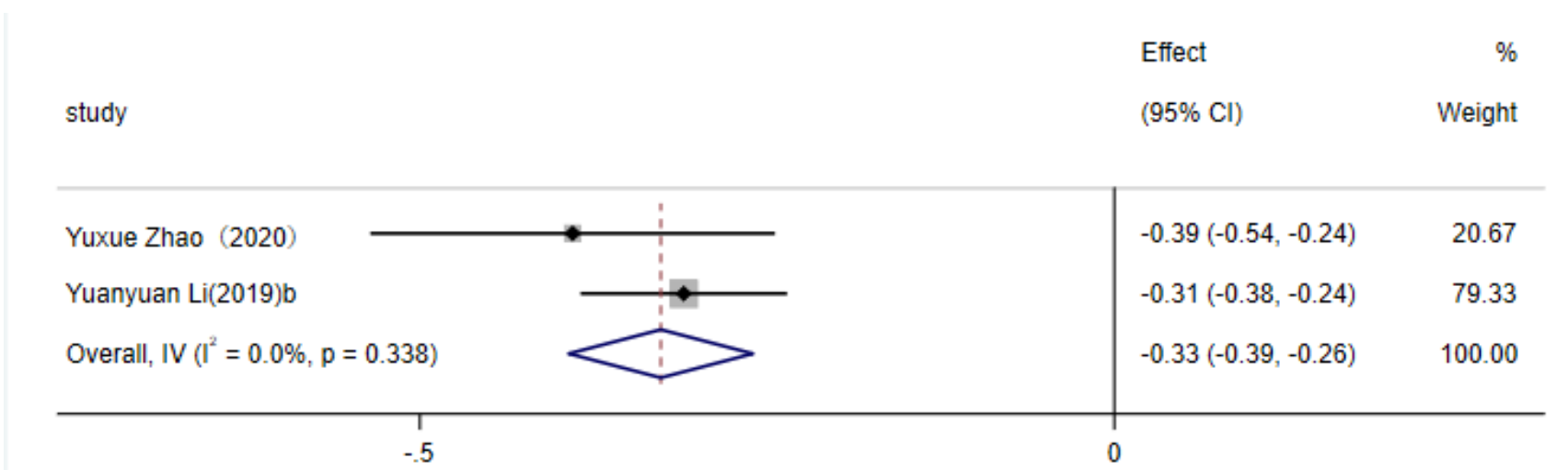

I. TLR4 Forest plot

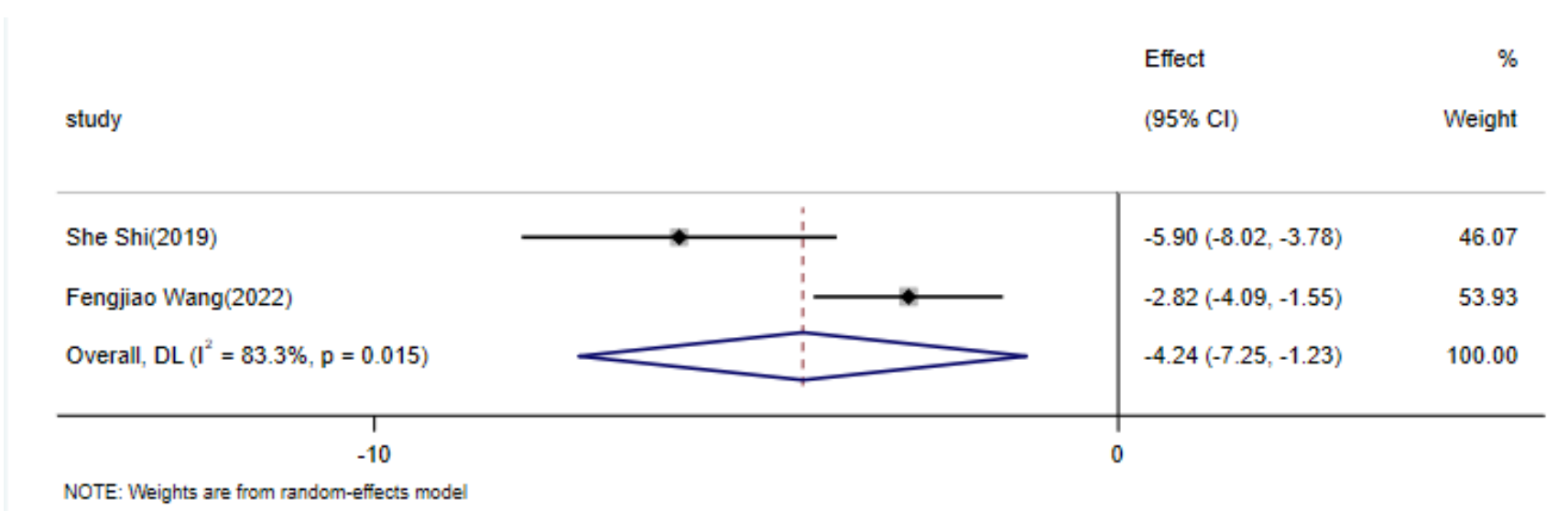

J. TRPA1 Forest plot

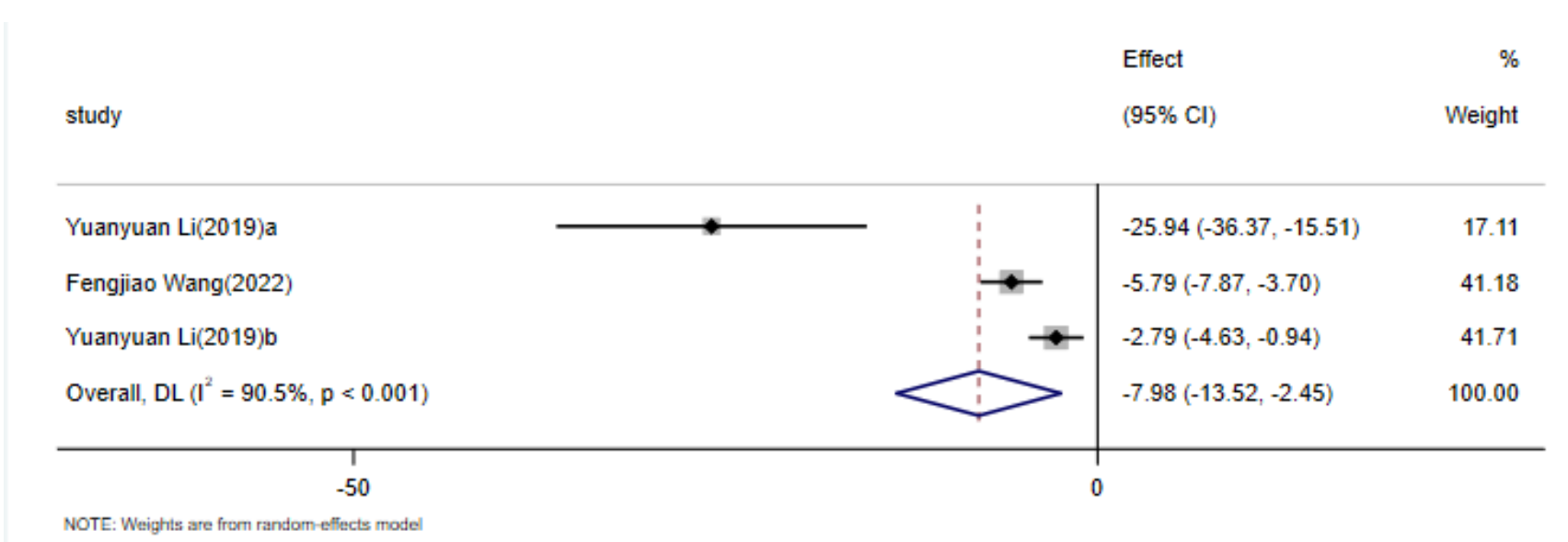

K. TRPV1 Forest plot

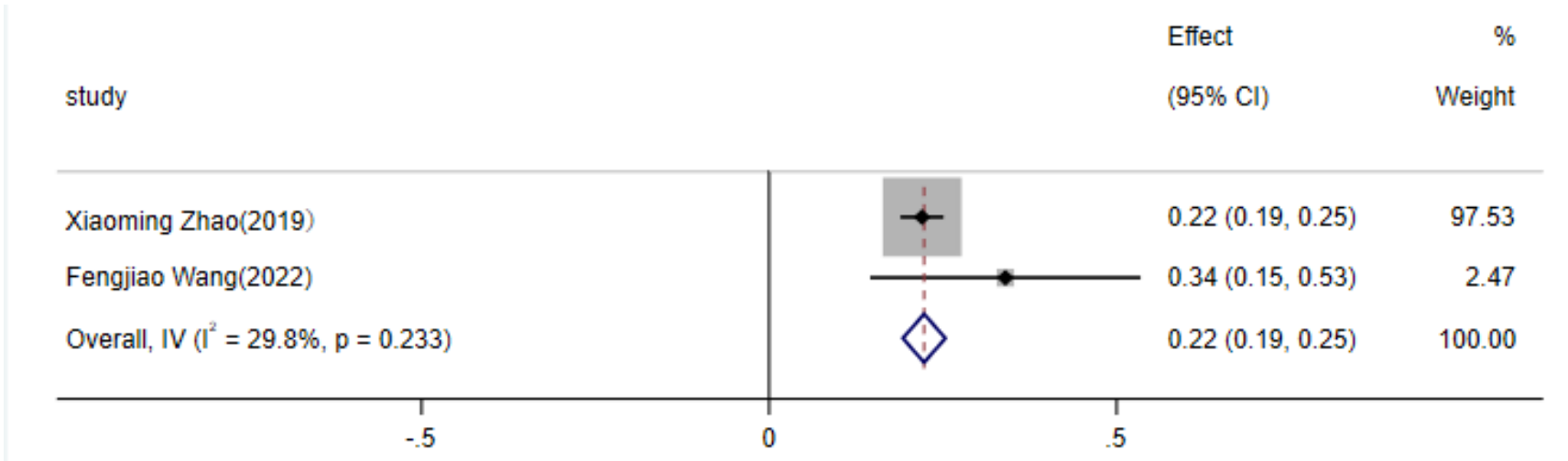

L. Nrf2 Forest plot
